# Steady-state statistics, emergent patterns and intermittent energy transfer in a ring of oscillators

**DOI:** 10.1007/s11071-022-07275-z

**Published:** 2022-02-18

**Authors:** Tiemo Pedergnana, Nicolas Noiray

**Affiliations:** CAPS Laboratory, Department of Mechanical and Process Engineering, ETH Zürich, Sonneggstrasse 3, 8092 Zürich, Switzerland

**Keywords:** Emergent patterns, van der Pol oscillator, Synchronization, Thermoacoustic instability, Fokker–Planck equation

## Abstract

Networks of coupled nonlinear oscillators model a broad class of physical, chemical and biological systems. Understanding emergent patterns in such networks is an ongoing effort with profound implications for different fields. In this work, we analytically and numerically study a symmetric ring of *N* coupled self-oscillators of van der Pol type under external stochastic forcing. The system is proposed as a model of the thermo- and aeroacoustic interactions of sound fields in rigid enclosures with compact source regions in a can-annular combustor. The oscillators are connected via linear resistive coupling with nonlinear saturation. After transforming the system to amplitude-phase coordinates, deterministic and stochastic averaging is performed to eliminate the fast oscillating terms. By projecting the potential of the slow-flow dynamics onto the phase-locked quasi-limit cycle solutions, we obtain a compact, low-order description of the (de-)synchronization transition for an arbitrary number of oscillators. The stationary probability density function of the state variables is derived from the Fokker–Planck equation, studied for varying parameter values and compared to time series simulations. We leverage our analysis to offer explanations for the intermittent energy transfer between Bloch waves observed in acoustic pressure spectrograms observed of real-world gas turbines.

## Introduction

### Thermoacoustic instabilities in can-annular combustors

In a rigid enclosed volume, the heat release rate response of an compact unsteady flame to acoustic perturbations forms a feedback loop with the acoustics of the enclosure. If, at a given condition, this interaction between the sound field and the flame exceeds the dissipation due to radiation losses and viscous effects in the fluid, occurring mainly at the boundary of the enclosure, it can give rise to so-called thermoacoustic instabilities. The study of this physical phenomenon goes back to the work of Rayleigh [1]. When an instability is insufficiently damped, it can lead to high-amplitude acoustic pressure oscillations in the enclosure. In industrial machines, such as stationary gas turbines, the resulting pulsations induce high-cycle fatigue cracks in the metal parts surrounding the enclosure, which cause down-time, incurring fees for the manufacturer, and can sometimes lead to catastrophic system failures if a broken part flies through the combustor and collides at high speed with the vanes and blades of the turbine. A review of high-cycle fatigue in gas turbines is given in Ref. [2]. Applied studies on Helmholtz dampers to suppress thermoacoustic instabilities in gas turbine combustors are found in Refs. [3, 4]. Suppression of a thermoacoustic instability in two coupled Rijke tubes in the presence of noise is discussed in Ref. [5]. Sensitivity and nonlinear dynamics of thermoacoustic oscillations are reviewed in [6]. A complex systems approach to modeling and mitigating thermoacoustic instability in turbulent combustors is presented in [7]. Synchronization phenomena and nonlinear energy pumping in two coupled thermoacoustic oscillators are studied experimentally in [8].

Since the turn of the millennium, the modeling, prediction and suppression of thermoacoustic instabilities have drawn renewed interest due to internationally imposed emission limits on power generation systems and the resulting increased demand for lean-premixed combustion. In a lean-premixed combustor, the entire air flow passes through the burner to ensure a sufficiently lean mixture and to avoid rich reactant pockets, which produce significantly more pollutants. There are two consequences to this concept: First, lean premixed flames are much more sensitive to flow perturbations than non-premixed flames, which increases critically their response to acoustic forcing. Secondly, in a premixed setting, one does not need to dilute the combustion products to temperatures that are acceptable for the available cooling of the turbine parts, and hence there are no dilution holes, which provide strong acoustic damping in non-premixed combustors. These two factors lead to combustion instability problems in lean-premixed gas turbines similar to those encountered in rocket engines. In both applications, these instabilities remain a key problem in the design process [9, 10].

Traditionally, most literature on modeling thermoacoustic instabilities focused on *silo-type* (one single can) or *annular* combustors (see, for instance, [11–14] and [15–17], respectively). However, as energy demand continues to rise and energy networks are changing, larger gas turbines are required. For these types of gas turbines, which produce more than 750 MW in combined cycles at more than $62\%$ efficiency, and which can be supplied with sustainably produced $\hbox {H}_2$ (hydrogen) that is blended with natural gas, the silo-type and annular combustor designs become uneconomical due to several engineering trade-offs. Therefore, modern high-efficiency H-class gas turbines exclusively feature *can-annular* combustor architectures. In this type of system, combustion takes place in a number (typically 12 or 16) of cans evenly distributed along the turbine annulus. The cans are thermodynamically decoupled from each other, but the annular turbine inlet is shared by all can outlets and allows for acoustic cross-talk between neighboring cans. Theoretical, numerical and experimental studies performed at Siemens [18–21], General Electric [22–24] and Ansaldo Energia Switzerland [25, 26] are testament to the increased interest of industry in the thermoacoustics of can-annular combustors. In Ref. [25], so-called Bloch modes, or rotating waves of the acoustic pressure field along the turbine annulus, are discussed. In Fig. 8 therein, the authors provide indirect experimental evidence of Bloch modes in a real-world gas turbine by decomposing measured acoustic pressure signals from different cans into rotating wave components of different azimuthal order using the discrete Fourier transform. Direct evidence of Bloch modes occurring in a four-can system, showing the synchronized state and a wave-like phase pattern along the annulus, is shown in Figs. 5 and 6 of Ref. [23], respectively. We note that the oscillators in the latter reference are coupled in a different way (by long tubes) than in the former reference (small gap near the turbine inlet), and that, as discussed in Sect. 1.4, our study focuses on a scenario closer to the setting of the former reference, which is typical for industrial can-annular gas turbines. The theoretical studies in Refs. [27, 28] use periodic (Bloch-type) boundary conditions to simplify the analysis of reduced-order models of can-annular combustors. The effect of resistive (damper-like) and reactive (mass- or spring-like) coupling on the linear stability of a ring of thermoacoustic oscillators is investigated in Ref. [29]. We also mention that nonlinear phenomena in can-annular configurations, such as amplitude death and quenching, have drawn increased interest in recent years [30–33].

### Aeroacoustic coupling between the cans

The cans are acoustically coupled through the apertures at the turbine inlet, leading to acoustic-hydrodynamic effects which are strongly influenced by the mean axial bulk flow speed of the combustion products $U_\mathrm {tot}$: When a sound wave with frequency *f* from inside the can is reflected at the coupling aperture of streamwise width *W*, the acoustic (compressible) velocity field inside the can interacts with the hydrodynamic (incompressible) fluid motion in the aperture. As a consequence of this interaction, depending on the value of the nondimensional Strouhal number $fW/U_\mathrm {tot}$, either destructive or constructive interference can occur.

**Fig. 1:**
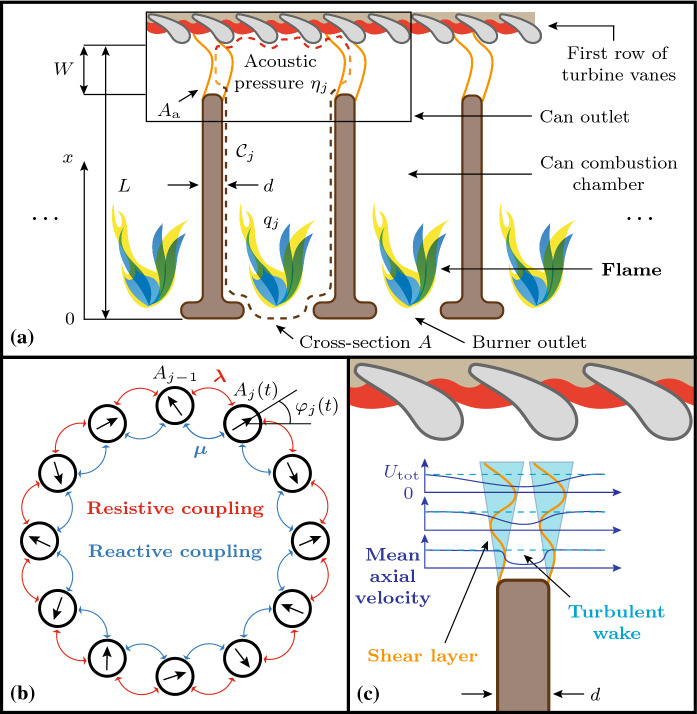
**a** Sketch of the modeled can-annular combustor, from a radial view. The dimensions are not true to scale. Shown are the control volume ${\mathcal {C}}_j$, the acoustic pressure at the can outlet $\eta _j$, the aperture width *W*, cross-section area $A_\mathrm {a}$ and depth *d*, respectively, the can length *L* and cross-section area *A*, respectively, as well as the coherent heat release fluctuations of the flame $q_j$, which characterize the flame response to acoustic perturbations. **b** Abstraction of the system illustrated in (**a**), viewed from an axial perspective, for $N=12$ cans. Shown are the amplitude $A_j$ and phase $\varphi _j$ of the acoustic pressure at the can outlet $\eta _j$, respectively, the linear resistive coupling $\lambda$ and the reactive coupling $\mu$. For simplicity, reactive coupling is neglected in this study. **c** Sketch of the turbulent wake in the aperture between neighboring cans. Shown is a typical profile of the mean axial velocity, the mean axial bulk flow speed of the combustion products $U_\mathrm {tot}$ and the turbulent wake, which is bounded by two shear layers. The acoustic-hydrodynamic interaction in the aperture leads to the resistive and reactive coupling between the oscillators shown in (**b**)

On the hydrodynamic side, the acoustic pressure fluctuations can lead to shear layer flapping in the turbulent wake between the cans (see Fig. 1c), which causes vorticity fluctuations that generate sound according to Howe’s acoustic energy corollary [34]. For a study on sound production by shear layer flapping in a laboratory-scale aeroacoustic system (a whistling beer bottle under grazing flow over the bottle top), the reader is referred to Ref. [35]. In extreme cases, at high pressure amplitudes, the shear layer can roll up and form discrete vortices which are shed periodically from the upstream edge of the aperture and produce sound when they meet the downstream edge. Numerical studies of sound generation by vortex shedding in side-branch apertures can be found in Refs. [36, 37].

The main consequence of the acoustic-hydrodynamic interaction described above is the following. For fixed aperture width *W* and frequency *f*, depending on the mean flow speed $U_\mathrm {tot}$, the sound field may lose or gain acoustic energy from the interaction with the grazing mean flow over the aperture. Experimental and numerical studies highlight also the role of the nonlinear saturation of the aeroacoustic response of a shear layer [38, 39]: When the acoustic forcing amplitude is increased, the response of the shear layer, in terms of its kinetic energy or its acoustic energy production at the fundamental frequency of excitation, decreases. A theoretical model of this nonlinear shear layer response, based on Howe’s formulation, was derived and validated against experiments in [40]. The same saturation mechanism leads to self-excited aeroacoustic instabilities, which occur, for example, when we whistle. The first modern study of this type of instability was conducted by Sondhauss in the nineteenth century [41]. Readers interested in the role of aeroacoustic instabilities in whistling and musical instruments are referred to the classic experiments of Wilson [42] as well as the more recent review of Fabre et al. [43]. Aeroacoustic instabilities can also occur in industrial machines, where they cause noise pollution and fatigue damage of components [44]. A discussion of the aeroacoustic instability mechanism from an industrial perspective, with a focus on mitigation measures and design rules, is found in [45].

### Literature review

The study of synchronization and rotating waves in rings of limit cycle oscillators is not novel. We give below a review of related analyses and discuss how our work differs from these previous efforts. A pair of coupled oscillators is a limiting case of a ring (see, for instance, [46–50]). For compactness, we do not include these works in the review below.

We find the first modern study of rings of coupled van der Pol oscillators in the work of Endo and Mori from the 1970s [51], wherein the authors classify the limit cycle solutions in a symmetric ring of a large number of oscillators with resistive coupling. Deterministic averaging is performed to identify limit cycle amplitudes. All limit cycle solutions are found to be linearly stable, and this finding is confirmed by experiments on an electrical circuit with four elements. Their study is continued in [52], where the effect of delay in the coupling term is studied. It is found that the addition of such a delay can lead to instabilities of certain modes, which is confirmed experimentally. A group-theoretical approach to the classification of phase-locked solutions in rings of coupled oscillators is taken in [53].

In Ref. [54], averaging, linear stability analysis, simulations and experiments are used to study the effect of differing parameters in members of rings of three and four oscillators. In [55], theory and experiments are combined to study the effect of external forcing and non-uniform parameter distribution on the synchronization of a four-member ring of van der Pol oscillators. Following up on their earlier work in Ref. [56], in [57], the authors study the effect of a non-uniform distribution of the reactive coupling parameter along the ring on the system stability by transforming the system into a Hill equation. They show that a harmonic variation of the reactive coupling parameter along the ring can affect the linear stability of limit cycles. We mention also Ref. [58], where it is shown that particular asymmetric parameter distributions can favor the stability of the perfectly symmetric, synchronized solution in a ring of nonidentical limit cycle oscillators.

An approach based on Floquet analysis of Hill’s equation is taken by [59] to study the stability of synchronized states in a large ring of van der Pol oscillators. Therein, the authors put emphasis on linking their results to intercellular dynamics in biology. Concepts from neuroinformatics are invoked in Ref. [60], focusing on synchronization phenomena in a ring of van der Pol oscillators coupled by time-varying resistors.

In Ref. [61], the authors begin by characterizing limit cycle solutions in a ring of (linearly) resistively coupled limit cycle oscillators. They perform a linear stability analysis and derive a quasi-potential (a candidate function for Lyapunov’s second method of stability [62]) to determine the nonlinear stability of these solutions. After a change to amplitude-phase coordinates, deterministic averaging is applied to eliminate the fast oscillating terms. Subsequently, external noise forcing is added to the phase equation to numerically investigate stochastic effects on the synchronization between the oscillators. Using a small-noise approximation, the authors obtain analytical formulas for the mean time of a state to leave an attractor basin. Because the phase noise is added phenomenologically after deterministic averaging is carried out on the original, fast system, it is unclear how Eqs. (11) and (12) relate to Eq. (1) in Ref. [61].

We note that instabilities of azimuthal waves in a discrete, ring-shaped fluid-dynamical system are also encountered, for instance, in Refs. [63–66], where theoretical and experimental methods are combined to study of the dynamics in a ring of bouncing droplets. Amplitude death and phase-flip bifurcations between in-phase and anti-phase synchronization in a system of coupled chemical oscillators are investigated in [67].

### Aim of this work

In this work, we combine analytical and numerical tools to study a low-order model of a can-annular combustor. Our goal is to gain a qualitative understanding of some of the system’s dynamic features that are independent of the number of cans *N*. In particular, we are interested in how, in the steady state, the amplitude and phase statistics of the acoustic pressure in the cans are related to the intermittent energy transfer between different Bloch modes observed in real-world gas turbines. To this end, we propose a symmetric model where all reactive effects of the flame response and the aeroacoustic coupling are neglected, and only resistive effects are taken into account. We show that this simple model is able to describe a wide variety of possible emergent patterns, including synchronization, rotating waves as well as quasi-steady superpositions of clockwise (CW) and counterclockwise (CCW) waves, and that it reproduces the intermittent energy transfer between different Bloch mode components of the acoustic pressure observed in real-world gas turbines. These patterns are characterized and differ from each other by the change of the phase of the acoustic pressure along the ring. For example, a wave-like pattern is described by a sinusoidal variation of the phase and the in-phase synchronized state by a uniform phase distribution. A more detailed discussion is given in the next section after the governing equations of the system have been derived.

A sketch of the physical system we consider is shown in Fig. 1a from a radial view. Shown are the control volume ${\mathcal {C}}_j$, the acoustic pressure at the can outlet $\eta _j$, the aperture width *W*, cross-section area $A_\mathrm {a}$ and depth *d*, respectively, the can length *L* and cross section area *A*, respectively, as well as the coherent (at the acoustic frequency) heat release fluctuations of the flame $q_j$, which characterize the flame response to acoustic perturbations. Figure 1b shows an abstraction of the same system for $N=12$ cans from an axial perspective, the amplitude $A_j$ and phase $\varphi _j$ of the acoustic pressure at the can outlet $\eta _j$, the linear resistive coupling $\lambda$ and the reactive coupling $\mu$. For simplicity, reactive coupling is neglected in this study. We use the convention that a positive increment in *j* implies a CW rotation by an angle of $2\pi /N$ along the ring, and that $j=1$ corresponds to the oscillator at the 12/*N* o’clock position. The turbulent wake in the aperture between neighboring cans is sketched in Fig. 1c. Shown is a typical profile of the mean axial velocity in the aperture, the mean axial bulk flow speed of the combustion products $U_\mathrm {tot}$ and the turbulent wake, which is bounded by two shear layers.

From an acoustic perspective, we restrict our analysis to the case of nearly closed, weakly coupled cavities whose area ratio $A_\mathrm {a}/A$ is smaller than the ratio of effective aperture depth to can length $(d+\sqrt{A_a})/L$, respectively: $A_a/A<(d+\sqrt{A_a})/L <1$. In this regime, the effects of the coupling can be studied as small perturbations of the dynamics of *N* decoupled cavities. Elementary first-principles calculations quantifying this limit case are provided in [29]. As discussed in this reference, anti-phase synchronization can occur also in a different setting when the cavities are strongly coupled. Therefore, when a phase pattern is referred to, for example, as the “anti-phase synchronized state,” this should not be understood in an exclusive sense, but in the context of the parameter range considered in this work. In other words, the presently considered coupling scenario is not the only situation in which periodic phase patterns along the ring can arise.

In our model, the flame drives a single natural (longitudinal) eigenmode $\psi _0$ of the can with corresponding eigenfrequency $\omega _0$. In a first approximation, we assume that the mode shape of $\psi _0$ is unperturbed by the thermo- and aeroacoustic interactions and that the acoustic pressure signal is close to harmonic. These are often reasonable assumptions in practice [68, 69]. Following the low-order modeling approach presented in the latter two references as well as Ref. [70], the thermoacoustic instability in each can is described by a van der Pol oscillator with linear growth rate $\nu$ and nonlinear saturation $\kappa$. For simplicity, all oscillators are assumed to be identical. For $\omega \approx \omega _0$, where $\omega =2\pi f$ and *f* is the frequency of oscillation, the acoustic pressure field $p_j$ in the *j*th can is approximated by the unimodal projection

1 $$\begin{aligned} p_j({x},t)\approx \eta _j(t)\psi _0({x}), \end{aligned}$$

where $\eta _j$ is the dominant modal amplitude. It is the dynamic variable of the van der Pol oscillator. Following [71], we assume a pressure antinode at the turbine inlet $\psi _0(L)=1$ (see Fig. 1a). In this case, $\eta _j$ is equivalent to the acoustic pressure $p_j$ at the can outlet.

The oscillators are driven by white noise, which represents broadband, quasi-random acoustic perturbations induced by turbulent fluctuations of the mean axial flow in the cans.

The thermoacoustic growth rate $\nu$ can be further decomposed into a sum of the acoustic gains from the flame response and dissipative losses at the boundary of the enclosure, each of which may be measured separately [72]. The parameter $\kappa$ describes the saturation of the coherent response of the flame to acoustic perturbations: for $\nu >0$, a small acoustic perturbation is amplified by the flame and grows, until the response saturates at a point where the acoustic gain from the flame equals the dissipative losses, and a limit cycle is reached.

The acoustic coupling is incorporated in the oscillator model by linear and van der Pol-type nonlinear resistive terms. The linear terms are analogous to damper and resistor elements in mechanical and electrical oscillators, respectively.

This linear resistive coupling describes the small-amplitude aeroacoustic response of the turbulent wake in the aperture to pressure differences between neighboring cans. Consistent with the low-order model of an aeroacoustic instability validated against experiments in Ref. [38], we include the nonlinear saturation of the aeroacoustic response with increasing pressure amplitude by adding a quadratic term in the pressure difference to the linear coupling $\lambda$. When the nonlinear coupling term is neglected, the governing equations of our model describe a network of nonlinear oscillators with linear coupling. Such systems are ubiquitous in classical mechanics [73–76], chemistry [77–79] and biology [80–82].

We go further than previous studies by accounting for nonlinearity in the (purely resistive) coupling, in combination with additive stochastic forcing of the fast variables, which is consistently included in the averaging procedure. To the authors’ knowledge, this case, which leads to novel and unexpected results, has not been investigated so far.

Due to the symmetry of the system considered in this work, we find that different emergent patterns (synchronized state, rotating waves, etc.) occur at similar amplitudes and are mainly distinguished by different phase dynamics. In regard to this, we mention Adler’s equation for phase injection locking [83–85] and Kuromoto’s classic model [86], which has become a paradigmatic example of phase synchronization in coupled oscillators [87–89].

### Overview

In Sect. 2, we present the model and derive the potential governing the amplitude and phase dynamics. In Sect. 3, numerical experiments on the deterministic, noise-free system are performed and analyzed. In Sect. 4, the steady-state statistics of the noise-driven system are derived and studied. Selected results and topics for future research are discussed in Sect. 5. In Sect. 6, we summarize our conclusions.

## Theoretical model

In an isolated can, the interaction of a single, dominant acoustic mode with the flame can be represented by a self-excited (van der Pol) oscillator [68–70]. Such low-order oscillator models of thermoacoustic instabilities in silo-type combustors are well-understood in terms of their accuracy compared to more detailed modeling approaches and experimental results (see, for instance, Fig. 17 in Ref. [90]). As shown in the latter reference, the linear growth rate $\nu$ and the nonlinear saturation $\kappa$ can be adjusted to obtain a qualitative approximation of the experimentally measured phase space spanned by the acoustic pressure *p* and its time derivative ${\dot{p}}$. Our model combines *N* of such oscillators to approximate the acoustic pressure dynamics in a can-annular combustor.

Following Ref. [38], we account for the aeroacoustic interaction between neighboring cans with the linear resistive coupling $\lambda$ and its nonlinear saturation $\vartheta$.

Stochastic forcing of the acoustic field by broadband noise production in the turbulent flow in the cans is modeled by additive, zero-mean white noise signals $\xi _j$ of equal intensity $\varGamma$ with the autocorrelation functions $\langle \xi _j \xi _{j,\tau }\rangle _{\mathbb {R}}=\int _{\mathbb {R}} \xi _j(t)\xi _j(t+\tau ) {d}t=\varGamma \delta (\tau )$, where $\langle \cdot \rangle _{\mathbb {R}}$ denotes the time integral over the real line $[-\infty ,\infty ]$ and $\delta$ is the Dirac delta function.

Based on the above discussion on low-order models of thermo- and aeroacoustic instabilities, we propose the following model of *N* coupled van der Pol oscillators for the acoustic pressure dynamics in the can-annular combustor:

2 $$\begin{aligned} \ddot{\eta }_{j}+\omega _{0} ^2{\eta }_j= & {} 2\nu {\dot{\eta }}_j-\kappa \eta _j^2{\dot{\eta }}_j+ \lambda ({\dot{\eta }}_{j+1}+{\dot{\eta }}_{j-1}-2{\dot{\eta }}_j)\nonumber \\&+\vartheta \big [({\eta }_{j+1}-{\eta }_j)^2({\dot{\eta }}_{j+1}-{\dot{\eta }}_j)\nonumber \\&+({\eta }_{j-1}-{\eta }_j)^2({\dot{\eta }}_{j-1}-{\dot{\eta }}_j)\big ]+\xi _j, \end{aligned}$$

for $j=1,\ldots ,N$ and $j+k\equiv \mathrm {mod}(j+k,N)$, $k\in {\mathbb {N}}$. The sign before $\vartheta$ is positive because the coupling is amplifying if $\lambda <0$ and we assume that as in Ref. [40], the constructive aeroacoustic coupling saturates at high amplitudes (monotonic decay of constructive feedback strength). For simplicity, this work focuses on situations where the nonlinear aeroacoustic coupling can be considered as a small perturbation of the thermoacoustic oscillations in the cans, so that the saturation associated with the thermoacoustic instability is significantly larger than the saturation of the aeroacoustic coupling: $\kappa \gg \vartheta$. We exclude the case of vanishing resistive coupling in our study, because a vanishing linear response $\lambda =0$ with nonzero saturation $\kappa \ne 0$ is not physical. The parameter values used in the numerical examples throughout this work are listed in Table 1. If multiple values are used, the default value is shown in bold. It is indicated below if values different from the default ones are used.

**Table 1:** Parameter values used in the numerical examples throughout this work. If multiple values are used, the default value is shown in bold

Parameter	Meaning	Value
$\omega _0/2\pi$	Frequency of dominant acoustic mode	255 Hz
$\nu$	Thermoacoustic growth rate	$\{\varvec{25},50\}$ $\hbox {s}^{-1}$
$\kappa$	Thermoacoustic saturation	0.48 $\hbox {s}^{-1}$ $\hbox {Pa}^{-2}$
$G=\kappa \varGamma /8|\nu |^2 \omega _0 ^2$	Normalized white noise intensity	$2.88\times \{\varvec{10^{-1}},10^1,10^3\}$
$\varLambda =2\lambda /\nu$	Normalized linear resistive coupling	$\in [-1,2]$
$K=16\vartheta /\kappa$	Normalized coupling nonlinearity	$\{0, 1/6, \varvec{1/3}\}$
*N*	Number of oscillators	$\{3,\varvec{12}$}
*b*	Bloch wavenumber	$\{0,\pm 1,\ldots ,\pm \mathrm {floor}(N/2)\}$
$\beta$	Continuous Bloch wavenumber	$\in [-N/2,N/2]$
$B=\pi \beta /N$	Normalized Bloch wavenumber	$\in [-\pi /2,\pi /2]$

We define the coordinate change to amplitude-phase variables $\{A_j,\varphi _j\}$, $j=1,\ldots ,N$, as follows:

3 $$\begin{aligned} A_j= & {} \sqrt{\eta _j^2+\left( {\dot{\eta }}_j/\omega _0\right) ^2}, \end{aligned}$$

4 $$\begin{aligned} \varphi _j= & {} -\arctan ({\dot{\eta }}_j/\omega _0,\eta _j)-\omega _0t, \end{aligned}$$

where $\eta _j=A_j\cos {(\varphi _j+\omega _0 t)}$, ${\dot{\eta }}_j=-\omega _0 A_j\sin {(\varphi _j+\omega _0 t) }$ and $\arctan (y,x)$ is the two-argument arctangent function. Assuming that **(A)** the variation of $A_j$ and $\varphi _j$ over an acoustic period is negligible and **(B)** the oscillators perform quasi-sinusoidal motion, deterministic and stochastic averaging (see Refs. [91, 92] and [93, 94], respectively) can be performed on Eq. (2) to eliminate the fast-oscillating terms. This averaging is done in Appendix A and yields the slow-flow dynamics in amplitude-phase form:

5 $$\begin{aligned} {\dot{A}}_j= & {} A_j\bigg (\nu -\frac{\kappa A_j^2}{8}\bigg )\nonumber \\&+\frac{\lambda }{2}\big [A_{j+1}\cos {(\varDelta _{j+1})}+A_{j-1}\nonumber \\&\cos {(\varDelta _{j})}-2A_j\big ]\nonumber \\&+\frac{\vartheta }{8}\bigg [(A_{j+1}^3+3A_j^2 A_{j+1})\cos (\varDelta _{j+1})-\nonumber \\&A_j A_{j+1}^2\cos (2\varDelta _{j+1})\nonumber \\&+(A_{j-1}^3+3A_j^2 A_{j-1})\cos (\varDelta _{j})\nonumber \\&-A_j A_{j-1}^2\cos (2\varDelta _{j})\nonumber \\&-2(A_j^3+A_j A_{j+1}^2+A_j A_{j-1}^2)\bigg ] +\frac{\varGamma }{4\omega _0^2 A_j}+\zeta _j \nonumber \\ \end{aligned}$$

and

6 $$\begin{aligned} {\dot{\varphi }}_j= & {} \frac{\lambda }{2}\left[ \frac{A_{j+1}}{A_j}\sin (\varDelta _{j+1})-\frac{A_{j-1}}{A_j}\sin (\varDelta _{j})\right] \nonumber \\&+\frac{\vartheta }{8}\bigg [\Big (A_j A_{j+1}+\frac{A_{j+1}^3}{A_j}\Big )\sin (\varDelta _{j+1})-A_{j+1}^2\nonumber \\&\sin (2\varDelta _{j+1})-\Big (A_j A_{j-1}+\frac{A_{j-1}^3}{A_j}\Big )\sin (\varDelta _{j})\nonumber \\&+A_{j-1}^2\sin (2\varDelta _{j})\bigg ]+\frac{\chi _j}{A_j}, \end{aligned}$$

for $j=1,\ldots ,N$, where $\varDelta _j=\varphi _j-\varphi _{j-1}$ is the phase difference and $\zeta _j$ and $\chi _j$ are 2*N* uncorrelated white noise sources with the same noise intensity $\varGamma /2\omega _0^2$, respectively. The above definitions of $\varphi _j$ and $\varDelta _j$ were used in the derivation of the slow-flow system, given by Eqs. (5) and (6). For the presentation of our results in Sects. 3 and 4, we set $\varphi _j\equiv \mathrm {mod}(\varphi _j,2\pi )$ and $\varDelta _j\equiv \mathrm {mod}(\varDelta _j+\pi ,2\pi )-\pi$. These operations, which leave Eqs. (5) and (6) unchanged, are equivalent to adding integer multiples of $\pm 2\pi$ to $\varphi _j$ and $\varDelta _j$ so that the sums lie in the domains $[0,2\pi ]$ and $[-\pi ,\pi ]$, respectively.

The slow flow system (5) and (6) is the main focus of this work. It has the form of a 2*N*-dimensional Langevin equation [95]:

7 $$\begin{aligned}&{\dot{A}}_j=-\frac{\partial }{\partial A_j}{\mathcal {V}}(A_m,\varphi _m)+\zeta _j, \end{aligned}$$

8 $$\begin{aligned}&{A_j{\dot{\varphi }}_j=-\frac{1}{A_j}\frac{\partial }{\partial \varphi _j}{\mathcal {V}}(A_m,\varphi _m)+\chi _j,} \end{aligned}$$

where $j,m=1,\ldots ,N$ and the potential ${\mathcal {V}}$ is defined as follows:

9 $$\begin{aligned}&{\mathcal {V}}(A_m,\varphi _m)=\sum _{l=1}^N \bigg (-\frac{\nu A_l^2}{2}+\frac{\kappa A_l^4}{32}-\frac{\lambda A_l}{2}\big [A_{l+1}\nonumber \\&\quad \cos (\varDelta _{l+1})-A_l\big ]-\frac{\vartheta A_l}{8}\Big [(A_{l+1}^3+A_l^2 A_{l+1})\cos (\varDelta _{l+1})\nonumber \\&\quad -\frac{A_l A_{l+1}^2}{2}\cos (2\varDelta _{l+1})-\frac{A_l}{2}(A_{l}^2+2A_{l+1}^2)\Big ] -\frac{\varGamma }{4\omega _0^2}\ln (A_l)\,\bigg ). \nonumber \\ \end{aligned}$$

Equations (7) and (8) imply that trajectories $(A_j,\varphi _j)$, $j=1,\ldots ,N$, are stochastically perturbed and attracted toward lower values of the potential ${\mathcal {V}}$ defined in Eq. (9).

In the deterministic case, trajectories of the fast and averaged systems are pointwise close to each other over time, and the degree of closeness is determined by how strongly the averaging assumptions **(A)** and **(B)** are satisfied [91, 92]. For a linear damped harmonic oscillator, the averaged system is equivalent to the fast system. In contrast to averaging in deterministic systems, due to the lack of smoothness, we do not expect trajectories of the stochastically averaged system to be pointwise close to those of the fast system, but for the joint probability density function (PDF) ${\mathcal {P}}(\eta _m,{\dot{\eta }}_m/\omega _0,t)$, $m=1,\ldots ,N$, computed from the fast and the averaged system, given by Eq. (2) and Eqs. (5) and (6), respectively, to converge in the steady state, i.e., for $t\rightarrow \infty$.

Several theorems on stochastic averaging exist [93, 94]. It is beyond the scope of this work to elaborate on these mathematically intricate results, which would require introducing additional notation and theory to understand under which conditions they apply to the fast system (2). Instead, it is accepted that stochastic averaging is justified by a combination of physical and mathematical arguments, and careful numerical validation is performed to ensure that, for the parameter range considered, the joint PDFs of the fast system (2) and the slow amplitude-phase system (5) and (6) indeed coincide over long time spans $[0,t_\mathrm {end}]$, $t_\mathrm {end}\gg \mathrm {max}(1/\nu ,\tau _{\xi _j},\tau _{A_j},\tau _{\varphi _j})$, where $1/\nu$ is the relaxation time ($\sim$ time is takes to reach the steady state) and $\tau _{a}$ is the correlation time of the signal “*a*”. The correlation time is defined as $\tau _a= \int ^{\infty }_{0^+} \langle \xi _j \xi _{j,\tau }\rangle _{\mathbb {R}} d\tau$, where $(\cdot )^+$ denotes the one-sided limit from above (see Ref. [93], p. 65, Eq. (3.61) with $\tau =t_2-t_1$). This validation ensures that any qualitative statistical observations from long time series of the averaged system carry over to the fast system.

We note that in real-world experiments and in numerical simulations, the signals $\xi _j$, $\zeta _j$ and $\chi _j$ will generally have nonzero correlation times. However, because we model $\xi _j$, $\zeta _j$ and $\chi _j$ as white noise signals with (theoretically) vanishing correlation times, we assume throughout this work that $1/\nu \gg \mathrm {max}(\tau _{\xi _j},\tau _{A_j},\tau _{\varphi _j})$, i.e., that the time to reach the steady state significantly exceeds the correlation time of the external stochastic forcing.

## Potential landscape of the deterministic system

In the absence of noise, $\varGamma =0$. Under the averaging assumptions **(A)** and **(B)**, the limit cycles of the deterministic part of the fast system (Eq. 2 with $\xi _j\equiv 0$) are given by fixed points $\{{\dot{A}}_j=0,{\dot{\varphi }}_j=0\}$ of the slow-flow system (5) and (6) with $\zeta _j\equiv \chi _j\equiv 0$. We find that these fixed points describe solutions with uniform amplitude along the ring and equal phase difference between neighboring oscillators:

10 $$\begin{aligned} A_j= & {} A_b^*, \end{aligned}$$

11 $$\begin{aligned} \varDelta _j= & {} \varDelta _b^*\quad \forall j. \end{aligned}$$

The periodicity condition $\varphi _j\equiv \varphi _{j+N}$ imposes the following additional restriction on the phase difference:

12 $$\begin{aligned} \varDelta _b^*=2\pi b/N,\quad b=0,\pm 1,\ldots ,\pm \mathrm {floor}(N/2). \end{aligned}$$

We call the solutions given by Eqs. (10)–(12) Bloch modes and *b* the Bloch wavenumber because of the present result’s similarity to Felix Bloch’s classic theory of electron dynamics in perfectly periodic crystal lattices [96]. For compactness, we also introduce the continuous and normalized Bloch wavenumbers $\beta \in [-N/2,N/2]$ and $B=\pi \beta /N\in [-\pi /2,\pi /2]$, respectively.

Solutions with $|b|>0$ appear as rotating waves along the ring [61]. We show below that under the current assumptions, these solutions are pairwise degenerate, i.e., that the linearization around the solutions with positive and negative *b* have the same exact eigenvalues. At the limit cycle, $\varphi _j=\mathrm {const.}\quad \forall j$ and $\eta _j(t)=A^*_b \cos (\omega _0 t+\varphi _1 + (j-1)\varDelta _b^*)$, so that, for $\varDelta _b^*>0$, under a negative increment in *j* (CCW shift along the ring) and a suitably chosen positive increment in *t*, the acoustic pressure $\eta$ remains constant. This implies that positive values of *b* ($\varDelta _b^*>0$) correspond to CCW rotating waves and negative values to CW rotating waves. In the absence of explicit asymmetries, both CW and CCW rotating waves have the same amplitude and frequency. The case $b=0$ is the in-phase synchronized solution, for which all oscillators reach maximum and minimum pressure simultaneously. For even *N*, we call the solution with $b=N/2$ the anti-phase synchronized solution (also referred to as the “push-pull mode” in thermoacoustics literature), which features a phase difference of $\pi$ between neighboring oscillators, so that neighboring oscillators reach maximum and minimum alternatingly.

We note that whereas the spinning direction the nodal lines of an apparent wave is determined by the sign of *b*, due to the finite number of members in the oscillator ring *N*, if $\mathrm {mod}(N,|b|)\ne 0$, there appear to be fewer nodal lines than expected (a Bloch mode with wavenumber *b* has exactly |*b*| nodal lines) and they appear to spin in the opposite direction than expected from the sign of *b*. This visual phenomenon is a consequence of a spatial analogue of the wagon-wheel effect [97] and is discussed more in Sect. 5.

For vanishing coupling $\lambda =\vartheta =0$, the system (2) corresponds to *N* decoupled van der Pol oscillators, whose weak-nonlinearity limit cycle solution $\{A_j\equiv \sqrt{8\nu /\kappa },\varphi _j=\mathrm {const.}\}$, $j=1,\ldots ,N$, is asymptotically stable [70]. By the hyperbolicity of this limit cycle and the implicit function theorem [98], linear stability persists for small enough coupling parameters $\{\lambda ,\vartheta \}$, which act as small perturbations of the decoupled system. Indeed, for small enough $\lambda$, all fixed points are linearly stable. This can be seen by linearizing Eqs. (5) and (6) around the fixed points defined in Eqs. (10)–(12) by writing $A_j\approx A_b^*+A'_j$ and $\varDelta _j\approx \varDelta _b^*+\varDelta '_j$, where $A'_j$ and $\varDelta '_j$ are small perturbations of the limit cycle amplitude (10) and the phase difference (12). Applying periodic boundary conditions to the linearization (see Refs. [25, 27, 28]) yields

13 $$\begin{aligned} {\dot{A}}_j'= & {} -2 [\nu -2\lambda \sin (\pi b/N)^2 ]A'_j, \end{aligned}$$

14 $$\begin{aligned} {\dot{\varphi }}_j'= & {} 0. \end{aligned}$$

This implies that the eigenvalues of the linearization of the system (5) and (6) around the degenerate rotating waves given by (10) and (12) are $\lambda _{1,2}=i\omega _0-2 [\nu -2\lambda \sin (\pi b/N)^2 ]$. For given *b* and $\lambda <\nu /2 \sin (\pi b/N)^2$, the linear stability of the corresponding limit cycles, demonstrated by Eq. (13), shows that all Bloch modes are local minima of the potential ${\mathcal {V}}$ given by Eq. (9). Therefore, for small enough linear resistive coupling $\lambda$, trajectories may converge to any of these solutions, dependent on their initial conditions.

**Fig. 2:**
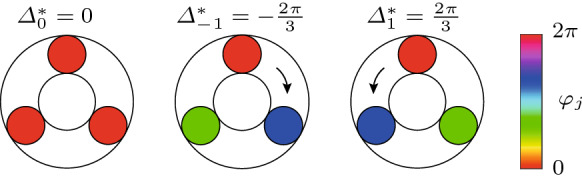
Illustration of the limit cycle solutions corresponding to $b=0$ and $b=\pm 1$ for $N=3$ oscillators. Shown are the values of the phase difference $\varDelta ^*_b$ and the phase distribution $\varphi _j$ along the ring. A positive value of *b* corresponds to a CCW rotating wave and a negative value to a CW rotating wave. The case $b=0$ is the synchronized solution, for which all oscillators are in phase

For $N=3$ oscillators, the limit cycle solutions corresponding to $b=0$ and $b=\pm 1$ are shown in Fig. 2. Shown are the values of the phase difference $\varDelta ^*_b$ and the phase distribution $\varphi _j$ along the ring.

Substituting Eqs. (10)–(12) into the slow-flow system (5) and (6) yields the limit cycle amplitudes

15 $$\begin{aligned} A^*_b=\sqrt{\frac{8\nu _b}{\kappa _b}}, \end{aligned}$$

where we have defined

16 $$\begin{aligned} \nu _b= & {} \nu -2\lambda \sin (\pi b/N)^2=\nu (1-\varLambda \sin ({B})^2), \end{aligned}$$

17 $$\begin{aligned} \kappa _b= & {} \kappa +16\vartheta \sin (\pi b/N)^4=\kappa (1+K \sin ({B})^4), \end{aligned}$$

$\varLambda =2\lambda /\nu$ is the normalized linear resistive coupling and $K=16\vartheta /\kappa$ the normalized coupling nonlinearity.

Limit cycles for a given value of the Bloch wavenumber *b* can only exist if

18 $$\begin{aligned} \lambda \le \frac{\nu }{2\sin (\pi b/N)^2}, \end{aligned}$$

otherwise the amplitude $A^*_b$ defined in Eq. (15) does not exist. Indeed, as evidenced by Eq. (13), when $\lambda$ exceeds this value for a given *b*, the corresponding rotating waves are linearly unstable. This occurs when the acoustic energy losses from the response to small amplitude perturbations of the turbulent wake in the apertures between the cans exceed the gain of acoustic energy from the coherent response of the flame.

In the following, we frequently make use of the assumption that all oscillators converge to quasi-limit cycles so that the dynamics take place in the vicinity of a two-dimensional submanifold $\mathrm {P}(\varOmega )$ of the 2*N*-dimensional phase space $\varOmega$, where

19 $$\begin{aligned} \varOmega =\{A_1,\varphi _1,\ldots ,A_N,\varphi _N\}, \end{aligned}$$

defined by $\mathrm {P}(\varOmega ):\quad A_j\equiv A^*_{\beta },\quad \varphi _j \equiv \varphi _{j-1}+k(j)\pi \beta /N\quad \forall j$, where $k(j)\in \{-1,1\}$. These quasi-limit cycle solutions include the case of true limit cycles, which are fixed points of the slow-flow system (5) and (6), for $k(j)\equiv 1$. However, we keep *k* general because, as we show below, quasi-steady solutions with uniform amplitudes $A_j\equiv A^*_{\beta }\approx \mathrm {const.}$ and uniform *absolute* phase differences $|\varDelta _j|\equiv |\varDelta _\beta ^*|=|\pi \beta /N|\approx \mathrm {const.}$ play a crucial role in the nonlinear dynamics of the oscillator ring. The projection of the potential ${\mathcal {V}}$ defined by Eq. (9) onto the submanifold $\mathrm {P}(\varOmega )$ reads

20 $$\begin{aligned} {\mathcal {V}}_\mathrm {P}= & {} {\mathcal {V}}(A_j\equiv A^*_\beta ,\varphi _j\equiv \varphi _{j-1}+k(j)\varDelta ^*_\beta )\nonumber \\= & {} -2N\nu _b \left|\frac{\nu _b}{\kappa _b}\right|. \end{aligned}$$

Normalizing this result, and using the fact that for a thermoacoustic instability, $\mathrm {sign}(\nu )=1$, yields

21 $$\begin{aligned} {\mathcal {U}}_\mathrm {P}(B)= & {} -(1-\varLambda \sin (B)^2) \left|\frac{1-\varLambda \sin (B)^2}{1+K\sin (B)^4}\right|, \end{aligned}$$

where ${\mathcal {U}}_\mathrm {P}=\kappa {\mathcal {V}}_\mathrm {P}/2N|\nu |^2$ is the normalized projection of the potential ${\mathcal {V}}$ onto the phase-locked quasi-limit cycle solutions. Note ${\mathcal {U}}_\mathrm {P}$ is independent of the signs in *k* and is symmetric with respect to positive and negative *B*. We show below that ${\mathcal {U}}_\mathrm {P}$ provides a compact description of the (de-)synchronization transition in the parameter space which is independent of the number of oscillators.

**Fig. 3:**
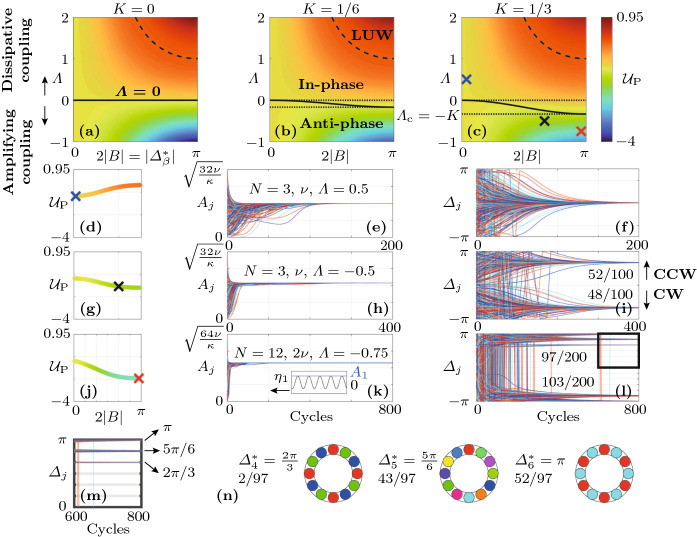
Numerical examples performed on the slow-flow system (5) and (6) in the deterministic limit with $\varGamma =0$. The distribution of the projected potential ${\mathcal {U}}_\mathrm {P}$, given by Eq. (21), as a function of $\varLambda$ and $2|B|=|\varDelta ^*_\beta |$ is shown in (**a**)–(**c**) for increasing values of the coupling nonlinearity *K*. The solid black curves parametrize the minima of ${\mathcal {U}}_\mathrm {P}$ with respect to 2*B* over $\varLambda$. The dotted lines bound the transition region, separating the domains where the in-phase (**In-phase**) and the anti-phase synchronized state (**Anti-phase**) are the global minimum of the potential ${\mathcal {V}}$, given by Eq. (9), respectively. Beyond the dashed black line are linearly unstable rotating waves (**LUW**). The blue, black and red crosses in Fig. **(c)** mark the location of the minimum of the projected potential ${\mathcal {U}}_\mathrm {P}$, given by Eq. (21), for $\varLambda =0.5$, $-0.5$ and $-0.75$, respectively. The variation of ${\mathcal {U}}_\mathrm {P}$ over 2|*B*| is shown in **(d)**, **(g)** and **(j)**. Time traces from random initial conditions of the amplitudes $A_j$ and phase differences $\varDelta _j$ are shown in **(e)**, **(f)** ($\varLambda =0.5$), **(h)** and **(i)** ($\varLambda =-0.5$) for $N=3$ oscillators (100 realizations) and in **(k)** and **(l)** for $N=12$ oscillators with $\varLambda =-0.75$ (200 realizations). The oscillators $j=1,2,3$ are indicated by blue, red and cyan color, respectively. In the small inset in **(k)**, the numerical solution $\eta _1$ (black) of the fast system (2) is compared with the amplitude $A_1$ (blue) computed from the the slow-flow system (5) and (6) for five cycles, starting at 200. The numbers above the trajectories in **(i)** and **(l)** indicate how many realizations converge to the solution classes with $\varDelta _j\lessgtr 0$ (CCW and CW rotating waves, respectively). The inset **(m)** shows an enlarged version of the time traces with $\varDelta _j>0$ in **(l)** between 600 and 800 cycles. In **(n)**, it is indicated how the 97 realizations in **(l)** with $b>0$ are distributed among different Bloch modes

The Taylor expansion of the projected potential ${\mathcal {U}}_\mathrm {P}$, given by Eq. (21), around $B=0$ (synchronized state) is

22 $$\begin{aligned} {\mathcal {U}}_\mathrm {P}(B)\approx -1 + 2 \varLambda B^2 + O(B^4), \end{aligned}$$

and the expansion around $B=\pm \pi /2$ is

23 $$\begin{aligned} {\mathcal {U}}_\mathrm {P}(B)\approx & {} -(\varLambda -1)^2/(K+1)\nonumber \\&+2(\varLambda -1)(\varLambda +K)(B\mp \pi /2)^2/(K+1)^2\nonumber \\&+O((B\mp \pi /2)^4). \end{aligned}$$

For $\varLambda >0$ (dissipative coupling) and $B\approx 0$, we consider Eq. (22), which shows that the global minimum of the potential ${\mathcal {V}}$ is at $B=0$, i.e., the synchronized state. Conversely, for $\varLambda <\varLambda _\mathrm {c}=-K$ (strong amplifying coupling) and $B\approx \pm \pi /2$, we consider Eq. (23) which shows the global minima of ${\mathcal {V}}$ are at $2B=\varDelta ^*_{\pm N/2}=\pm \pi$ (or $\varDelta ^*_{\pm \mathrm {floor}(N/2)}$ for odd *N*), corresponding to anti-phase synchronization.

For $0>\varLambda >\varLambda _\mathrm {c}$, which we call the “transition region” in the following, the minima of the projected potential ${\mathcal {U}}_\mathrm {P}$, given by Eq. (21), lie between $B=0$ and $B=\pm \pi /2$. This parameter range is further investigated below. By computing the zero level set of the slope of ${\mathcal {U}}_\mathrm {P}$ with respect to *B*, we obtain the curve parameterizing the minima of ${\mathcal {U}}_\mathrm {P}$ in the transition region as a function of $\varLambda$:

24 $$\begin{aligned} 0&{=}\frac{\partial {\mathcal {U}}_\mathrm {P}}{\partial B}=2\bigg (\frac{\varLambda \sin (B)\cos (B)}{1-\varLambda \sin (B)^2}\nonumber \\&+\frac{2K\sin (B)^3\cos (B)}{|1+K\sin (B)^4|^2}\nonumber \\&\times (1+K\sin (B)^4)+\frac{\varLambda \sin (B)\cos (B)}{|1-\varLambda \sin (B)^2|}\bigg )(1-\varLambda \sin (B)^2)\nonumber \\&\quad \left|\frac{1-\varLambda \sin (B)^2}{1+K\sin (B)^4}\right|. \end{aligned}$$

**Fig. 4:**
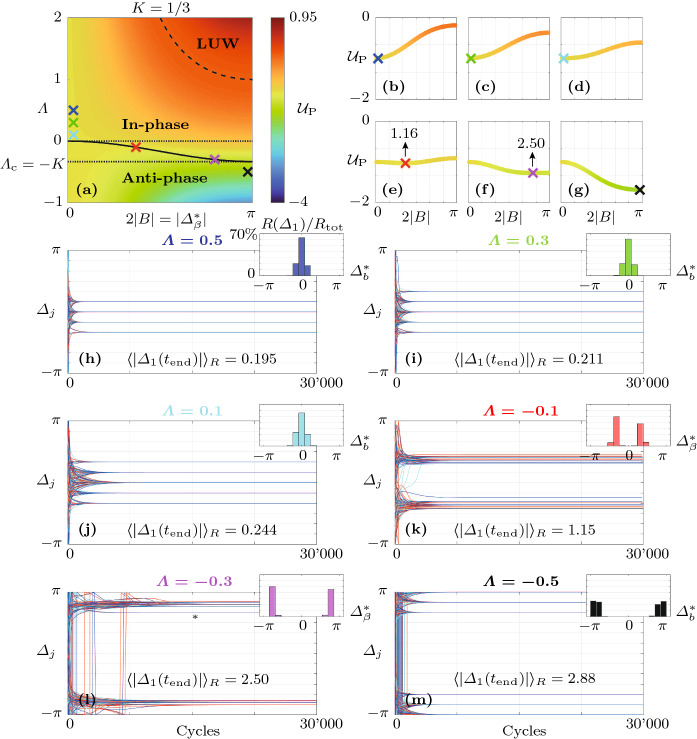
Numerical experiments near and in the transition region, which separates the two domains where the in-phase (**In-phase**) and the anti-phase synchronized state (**Anti-phase**) are the global minimum of the potential ${\mathcal {V}}$, given by Eq. (9), respectively, for an oscillator ring with $N=12$ members. **a** Distribution of the projected potential ${\mathcal {U}}_\mathrm {P}$, given by Eq. (21), as a function of $\varLambda$ and $2|B|=|\varDelta ^*_\beta |$. The colored crosses indicate the location of the minimum of the projected potential ${\mathcal {U}}_\mathrm {P}$, given by Eq. (21), over *B* for six evenly spaced values of the linear resistive coupling $\varLambda$, respectively. The corresponding distributions of the projected potential ${\mathcal {U}}_\mathrm {P}$ over the phase difference 2|*B*| is shown in (**b**)–(**g**). The positions of the minima of ${\mathcal {U}}_\mathrm {P}$ are indicated in (**e**) and (**f**), where they do not correspond to discrete Bloch modes. For each value of $\varLambda$, 500 realizations of the slow-flow system (5) and (6) were computed over 5000 cycles from random initial conditions. In (**h**)–(**m**), the phase differences $\varDelta _j$, $j=1,2,3$, are shown in blue, red and cyan, respectively. Time traces of other oscillators appear similar. In (**h**), (**i**), (**j**) and (**m**), the first 20 realizations converging to each Bloch mode are shown and in (**k**) and (**l**) the first 50 of all realizations are shown. The average of $|\varDelta _1|$ over all realizations at 5000 cycles, $\langle |\varDelta _1(t_\mathrm {end})| \rangle _R$, is displayed in the same insets. In (**h**)–(**m**), the smaller insets in the upper right corners show the relative share $R(\varDelta _1)/R_\mathrm {tot}$ of the total realizations $R_\mathrm {tot}$ of $\varDelta _1$ that fall into the bins corresponding to the different Bloch modes. Note that while in (**h**)–(**j**) and (**m**), all realizations converge to discrete Bloch modes, in (**k**) and (**l**) (transition region), trajectories generally ($100\%$ and $95\%$, respectively) converge to unsteady quasi-limit cycles which are superpositions of CW and CCW rotating waves, see Fig. 5. However, Bloch modes are also observed in the transition region; see, for example, the time trace marked with an asterisk (${}^*$) in **(l)**, which corresponds to $b=4$

Numerical examples were performed and analyzed to explore the projected potential ${\mathcal {U}}_\mathrm {P}$, given by Eq. (21), by launching a number of realizations of the slow-flow system (5) and (6) from random initial conditions. A part of the results is reported in Fig. 3, where, for $N=3$ oscillators and $\nu =25$ $\hbox {s}^{-1}$, $R=100$ realizations were computed for $\varLambda =\pm 0.5$, respectively, and $R=200$ realizations were computed for $N=12$ oscillators, $\nu =50$ $\hbox {s}^{-1}$ and $\varLambda =-0.75$. Trajectories of the oscillators $j=1,2$ and 3 are shown in blue, red and cyan, respectively. For $N=12$, only the first three oscillators are shown, but trajectories of other oscillators appear similar. In Fig. 3a–c, the distribution of the projected potential ${\mathcal {U}}_\mathrm {P}$ as a function of $\varLambda$ and $2|B|=|\varDelta _b^*|$ is shown. These insets illustrate how a nonzero coupling nonlinearity $K>0$ leads to a transition region for $0>\varLambda >\varLambda _\mathrm {c}=-K$, bounded by the dotted lines, which separates the domains where the in-phase (**In-phase**) and the anti-phase synchronized state (**Anti-phase**) are the global minimum of the potential ${\mathcal {V}}$, given by Eq. (9), respectively. The solid black curve indicates the location of the minimum of ${\mathcal {U}}_\mathrm {P}$ with respect to 2*B* in the transition region. The blue, black and red crosses in 3c mark the location of the minimum of the projected potential ${\mathcal {U}}_\mathrm {P}$, given by Eq. (21), for $\varLambda =0.5$, $-0.5$ and $-0.75$, respectively. The dashed black curve demarcates the domain within which Bloch modes are linearly unstable (**LUW**: linearly unstable waves). Figure 3d, g and j shows the variation of the projected potential ${\mathcal {U}}_\mathrm {P}$ as a function of 2|*B*|. In Fig. 3e, f, ($\varLambda =0.5$), h, i ($\varLambda =-0.5$), it is shown how, in a ring of $N=3$ oscillators with dissipative coupling, all trajectories converge to the synchronized state and for strong amplifying coupling with $\varLambda <\varLambda _\mathrm {c}=-K$, all trajectories converge to rotating wave solutions, with a symmetric distribution between CW ($\varDelta _j<0$) and CCW ($\varDelta _j>0$) rotating waves. The number of realizations that converge to either solution class is indicated above the $\varDelta _j$-curves. As shown in Fig. 3k, l, for $N=12$ and $\varLambda =-0.75$, out of all trajectories that converge to solutions with positive *b* (97 out of 200 total realizations), the majority (52 out of 97) converge to the limit cycle with $b=N/2=6$, but a significant amount of them do not, and instead converge to the nearby solutions with $b=5$ (43 out of 97) and $b=4$ (2 out of 97). The small inset in Fig. 3k shows a comparison between a single realization of $\eta _1$ (black) computed from the fast system (2) and the amplitude $A_1$ (blue) computed from the slow-flow system (5) and (6) for 5 cycles, starting at 200. Figure 3m shows an enlarged version of Fig. 3l with $\varDelta _j>0$ between 600 and 800 cycles. The corresponding Bloch modes are visualized in Fig. 3n. Similar aggregating behavior around the synchronized state with $b=0$ is observed for $\varLambda >0$, see Fig. 4.

Figure 3 shows that while a change in $\varLambda$ from 0.5 to $-0.5$ leads to *O*(1) changes in the phase differences $\varDelta _j$, the corresponding change in the amplitudes $A_j$ in the steady state is small compared to $A_0^*$. To better understand this, note that on the one hand, for $\varLambda >0$, the synchronized state is globally attracting, and the system aggregates around low-order Bloch modes $b\approx 0$, which implies a small influence of $\varLambda$ and *K* on the limit cycle amplitude $A_b^*$, see Eqs. (15)–(17). On the other hand, for $\varLambda <0$ and $0<K\ll 1$, we have $(A_b^*)^2\approx (A_0^*)^2(1-\varLambda \sin (\pi b/N)^2-K\sin (\pi b/N)^4)$, i.e., $\varLambda$ and *K* tend to cancel each other out. For this reason, and because the aeroacoustic coupling is considered as a small perturbation of the thermoacoustic limit cycles ($|\varLambda |\ll 1$, $K\ll 1$), the coupling-induced changes in the amplitude are in general assumed to be small. For $\varLambda <\varLambda _\mathrm {c}=-K$, solutions with $|b|>0$ reach limit cycle amplitudes $A_b^*$ exceeding that of the synchronized state: $A_b^*>A_0^*$. However, such small variations of the oscillation amplitude are difficult to quantify in experiments on real aero- and thermoacoustic systems, which are typically plagued by significant noise levels. Accordingly, the present work mostly focuses on the phase differences $\varDelta _j$, whose distribution quantifies emergent patters of the acoustic pressure $\eta _j$ along the ring.

**Fig. 5:**
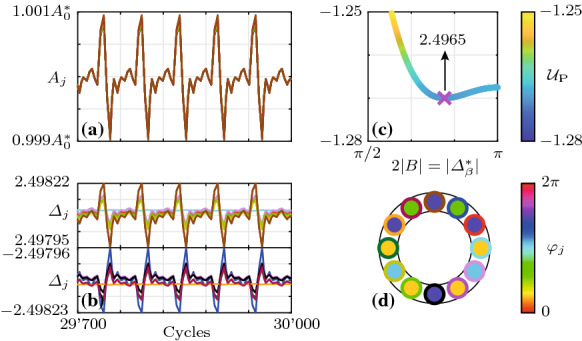
Example of a transition mode in the steady state, computed from the slow-flow system (5) and (6) for $\varLambda =-0.3$ ($\varLambda _\mathrm {c}=-0.{\overline{3}}$). Shown is **a** the variation of the amplitudes $A_j$ and **b** the phase differences $\varDelta _j\equiv \mathrm {mod}(\varphi _j-\varphi _{j-1}+\pi ,2\pi )-\pi$ for a single realization over 300 cycles in the steady state, **c** the distribution of the projected potential ${\mathcal {U}}_\mathrm {P}$ over $2|B|=|\varDelta _\beta ^*|$ (magnified view of Fig. 4**f** with a different colormap) and **d** the phases $\varphi _j$ at 30,000 cycles. The colors of the time traces in (**a**) and (**b**) correspond to those of the small rings in (**d**). Time traces of other transition modes appear similar, with more or less pronounced asymmetry between different oscillators, but with absolute phase differences $|\varDelta _j|$ near the minimum of ${\mathcal {U}}_\mathrm {P}$ over 2|*B*| (see Fig. 4k ,l). The phase distribution $\varphi _j$ along the ring shown in (**c**) corresponds to a standing wave-type pattern of the acoustic pressure $\eta _j$ along the ring. Animated examples of transition mode time traces are contained in the Supplementary Material

More numerical experiments were performed on the slow-flow system (5) and (6) to explore the transition region. The corresponding results are shown in Fig. 4. Figure 4a shows the projected potential ${\mathcal {U}}_\mathrm {P}$, given by Eq. (21), as a function of $\varLambda$ and $2|B|=|\varDelta _\beta ^*|$. The colored crosses in 4a mark the location of the minimum of the projected potential ${\mathcal {U}}_\mathrm {P}$, given by Eq. (21), over *B* for six evenly spaced values of the linear resistive coupling $\varLambda$, respectively. For the same values of $\varLambda$, the insets in Fig. 4b–g show the distributions of ${\mathcal {U}}_\mathrm {P}$ over the phase difference 2|*B*|. The positions of the minima of ${\mathcal {U}}_\mathrm {P}$ are indicated in Fig. 4e, f, where they do not correspond to discrete Bloch modes. Five hundred realizations of the slow-flow system (5) and (6) were computed over 5000 cycles from random initial conditions at each value of $\varLambda$. The insets in Fig. 4h–m show the evolution of the phase differences $\varDelta _j$ of the oscillators with $j=1,2$ and 3 in blue, red and cyan, respectively. In Fig. 4h–j, m, the first 20 realizations converging to each Bloch mode are shown, and in Fig. 4k ,l, the first 50 of all realizations are shown. The average of $|\varDelta _1|$ over all realizations at 5000 cycles, $\langle |\varDelta _1(t_\mathrm {end})| \rangle _R$, is displayed in the same insets. In Fig. 4h–m, the smaller insets in the upper right corner show the relative share $R(\varDelta _1)/R_\mathrm {tot}$ of the total realizations $R_\mathrm {tot}$ of $\varDelta _1$ that fall into the bins corresponding to the different Bloch modes. Note that while in Fig. 4h–j, m, all realizations converge to discrete Bloch modes, in Fig. 4k, l (transition region), trajectories generally ($100\%$ and $95\%$, respectively) converge to unsteady quasi-limit cycles which we call “transition modes.” These solutions are superpositions of CW and CCW rotating waves, see Fig. 5. Consistent with the linear stability analysis performed above, in the transition region, Bloch modes are also observed; see, for example, the time trace marked with an asterisk (${}^*$) in Fig. 4l, which corresponds to $b=4$.

We observe in Fig. 4 that outside the transition region, for $\varLambda >0$ or $\varLambda <\varLambda _\mathrm {c}$, when the global minimum of the potential ${\mathcal {V}}$, given by Eq. (9), coincides with a fixed point of the slow-flow system (5) and (6), all trajectories converge to discrete Bloch modes. For $\varLambda >0$ and $\varLambda <0$, nearly symmetric unimodal and bimodal distributions of the phase difference $\varDelta _1$ over $\varDelta _b^*$ and $\varDelta _\beta ^*$ are observed in the numerical experiments, respectively.

Inside the transition region, for $0>\varLambda >\varLambda _\mathrm {c}$, we observed in our numerical experiments that trajectories generally do not converge to the phase-locked fixed points of the slow-flow system (5) and (6), but to unsteady quasi-limit cycles, the transition modes, which feature small-scale, piecewise linear periodic variation of the amplitudes $A_j$ and phase differences $\varDelta _j$’. In the transition region, the mean $\langle |\varDelta _1(t_\mathrm {end})|\rangle _R$ over all realizations matches well with the minimum of the projected potential ${\mathcal {U}}_\mathrm {P}$ over $|\varDelta ^*_\beta |$, see Fig. 4e, f, k and I.

An example of a transition mode in the steady state, computed from the slow-flow system (5) and (6) for $\varLambda =-0.3$ ($\varLambda _\mathrm {c}=-0.{\overline{3}}$), is shown in Fig. 5. Shown in Fig. 5a, b are the variation of the amplitudes $A_j$ and the phase differences $\varDelta _j=\mathrm {mod}(\varphi _j-\varphi _{j-1}+\pi ,2\pi )-\pi$ for $j=1,\ldots ,N$, respectively, for a single realization over 300 cycles in the steady state. Figure 5c, d shows the distribution of the projected potential ${\mathcal {U}}_\mathrm {P}$ over $2|B|=|\varDelta _\beta ^*|$ (magnified view of Fig. 4f with a different colormap) and the phases $\varphi _j$ along the ring at the end time, respectively. The colors of the time traces in Fig. 5a, b correspond to those of the small rings in Fig. 5d. The phase differences $\varDelta _j$ perform slow, small-scale periodic motion over time. Because the transition modes feature both positive and negative phase differences $\varDelta _j$, these solutions are superpositions of CW and CCW rotating waves which appear as standing wave-type patterns of the acoustic pressure $\eta _j$ along the ring. After a transient, the amplitudes $A_j$ all remain quasi-steady, performing small-scale, periodic motion around $A_0^*$. Time traces of other transition modes appear similar, with more or less pronounced asymmetry between different oscillators, but with absolute phase differences $|\varDelta _j|$ near the minimum of ${\mathcal {U}}_\mathrm {P}$ over 2|*B*|, see Fig. 4k, l.

Animated examples of transition mode time traces ($A_j$, $\varphi _j$, $\varDelta _j$ and $\eta _j$), computed from the slow-flow system (5) and (6), are contained in the SupplementaryMaterial.

The numerical results reported in Figs. 4 and 5 suggest that in the transition region, the occurrence of quasi-limit cycles near the minimum of the projected potential ${\mathcal {U}}_\mathrm {P}$, given by Eq. (21), is generic. Despite these solutions not being fixed points of the slow-flow system (5) and (6), through this unsteady motion, the system reaches lower values of the potential ${\mathcal {V}}$, given by Eq. (9), and hence the transition modes are typically favored over the discrete Bloch modes in the transition region. Further research is required to explore the dynamic nature of these transition modes. Open topics are discussed in Sect. 5.

To better understand the transition modes’ unsteadiness, we consider the continuous Bloch wavenumber $\beta$ and assume the system reaches a quasi-limit cycle where $A_j\equiv A^*_\beta$, $\varDelta _j \equiv {k(j)} \varDelta ^*_\beta$, $k(j)\in \{-1,1\}$ and $j=1,\ldots ,N$. Of course this solution can never be exactly observed, because these quasi-limit cycles are not fixed points of the deterministic part of the slow-flow system given by Eqs. (5) and (6). The normalized potential ${\mathcal {U}}_\mathrm {P}$ is independent of the signs in *k*, see Eq. (21). However, the signs do impact the projection of the slow-flow equations (5) and (6) onto the quasi-limit cycle solutions $\{A_j\equiv A^*_\beta ,\varDelta _j\equiv k(j) \varDelta ^*_\beta \}$:

25 $$\begin{aligned}&{\dot{A}}_\beta ^*=\nu _\beta A_\beta ^*-\frac{\kappa _\beta (A_\beta ^*)^3}{8} \end{aligned}$$

26 $$\begin{aligned}&k(j){\dot{\varDelta }}^*_\beta =\frac{\lambda \sin (\varDelta ^*_\beta )}{2}\left[ {k(j+1)}-{k(j)}\right] \nonumber \\&\qquad \qquad \quad +\,\frac{\vartheta (A_\beta ^*)^2}{8} \bigg (2\sin (\varDelta ^*_\beta )\Big [{k(j+1)}-{k(j)}]\Big ]\nonumber \\&\qquad \qquad \quad -\sin (2\varDelta ^*_\beta )\Big [k(j+1)-k(j)\Big ]\bigg ), \end{aligned}$$

where the fact that in the steady state, ${\dot{\varphi }}_j\approx 0$ was used and $\nu _\beta$, $\kappa _\beta$ are obtained by replacing *b* with $\beta$ in Eqs. (16) and (17), respectively. For a distribution of $\varDelta _j$ with varying signs in *k* along the ring, Eq. (26) implies unsteadiness of the Bloch wavenumber $\beta$. This unsteadiness carries over to the equation for ${\dot{A}}_\beta ^*$ through $\nu _\beta$ and $\kappa _\beta$. The ad hoc argument made above to explain the transition modes’ unsteadiness is not an exact description of the nonlinear dynamics in the phase space $\varOmega$ (see Eq. 19), which are responsible for the occurrence of transition modes and require further investigation.

**Fig. 6:**
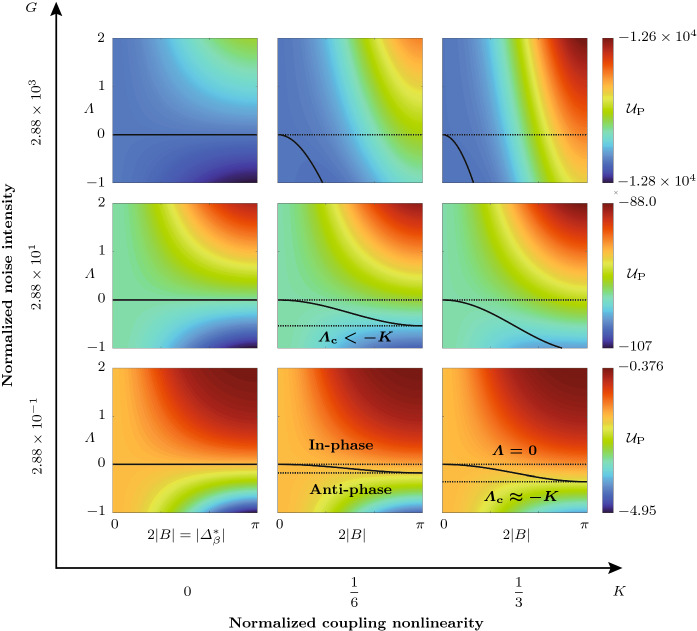
Distribution of the projected potential ${\mathcal {U}}_\mathrm {P}$, given by Eq. (28), as a function of $\varLambda$ and $2|B|=|\varDelta _\beta ^*|$ for varying noise intensity *G* and coupling nonlinearity *K*. The dashed black lines mark the transition region $0>\varLambda >\varLambda _\mathrm {c}$. In the bottom right inset, $\varLambda _\mathrm {c}=-0.356$. Above and below the transition region, the in-phase and the anti-phase synchronized state are the global minimum of the potential ${\mathcal {V}}$, given by Eq. (9), respectively. In the transition region, the solid black curve, defined by $\partial {\mathcal {U}}_\mathrm {P}/\partial B=0$, parametrizes the minima of ${\mathcal {U}}_\mathrm {P}$ with respect to 2*B* over $\varLambda$

In this section, the potential landscape of the deterministic system was investigated. To this end, the potential ${\mathcal {V}}$ of the amplitude-phase system (5) and (6) was projected onto the phase-locked, uniform-amplitude quasi-limit cycle solutions to obtain the normalized projected potential ${\mathcal {U}}_\mathrm {P}$, which compactly describes the (de-)synchronization transition, dividing the parameter space into two domains where the in-phase and the anti-phase synchronized state are the global minimum of the potential ${\mathcal {V}}$, respectively. These domains are separated by a transition region, whose size is proportional to the coupling nonlinearity *K* and which vanishes for $K=0$. Notably, ${\mathcal {U}}_\mathrm {P}$ is both non-dimensional and independent of the number of oscillators *N*.

## Steady-state statistics of the noise-driven system

Building on our analysis of the projected potential ${\mathcal {U}}_\mathrm {P}$ in the previous section, we begin our investigation of the noise-driven system by studying the behavior of ${\mathcal {U}}_\mathrm {P}$ under varying coupling nonlinearity *K* and noise intensity $\varGamma$. For $\varGamma \ne 0$, the fixed points of the deterministic part of the slow-flow system (5) and (6) are still characterized by uniform amplitudes along the ring and constant phase differences between neighboring oscillators. The phase differences are still defined by Eq. (12), while the limit cycle amplitudes are affected by the noise intensity $\varGamma$:

27 $$\begin{aligned} A^*_b=2\sqrt{\frac{\nu _b+\sqrt{\nu _b^2+\kappa _b\varGamma /8\omega _0^2}}{\kappa _b}}. \end{aligned}$$

In the case $\varGamma \ne 0$, the trajectories of the slow-flow system are governed by a gradient system perturbed by white noise forcing, described by Eqs. (7) and (8). Similar to the previous section, it is assumed that in the steady state, each oscillator performs stochastically fluctuating motion near the quasi-limit cycles of the deterministic system with uniform amplitude $A_j\equiv A^*_\beta$ and absolute phase differences $|\varDelta _j|\equiv |\varDelta ^*_\beta |$. The normalized projected potential ${\mathcal {U}}_\mathrm {P}$ depends now also on the normalized noise intensity $G=\kappa \varGamma /8|\nu |^2 \omega _0 ^2$ and is computed for different values of *K* and *G* by substituting the limit cycle amplitude (27) and $\varDelta _j\equiv \varDelta _\beta ^*$ into the potential ${\mathcal {V}}$, given by Eq. (9), and dividing the result by the factor $2N|\nu |^2/\kappa$ for normalization. For compactness, the resulting expression is only listed implicitly here:

28 $$\begin{aligned}&{\mathcal {U}}_\mathrm {P}(B)\nonumber \\&\quad =\frac{\kappa }{2N|\nu |^2}{\mathcal {V}}(A_j\equiv A^*_\beta ,\varphi _j\equiv \varphi _{j-1}+k(j)\varDelta ^*_\beta ) . \end{aligned}$$

where $A^*_\beta$ and $\varDelta ^*_\beta$ are obtained by replacing *b* with $\beta$ in Eqs. (12) and (27), respectively.

The distribution of the projected potential ${\mathcal {U}}_\mathrm {P}$ as a function of $\varLambda$ and $2|B|=|\varDelta ^*_\beta |$ is studied in Fig. 6. The dashed black lines mark the transition region $0>\varLambda >\varLambda _\mathrm {c}$. Note that $\varLambda _\mathrm {c}$ depends now on both *K* and *G*. Above and below the transition region, the in-phase and the anti-phase synchronized state are the global minimum of the potential ${\mathcal {V}}$, given by Eq. (9), respectively. The solid black curves in the transition region parametrize the minima of ${\mathcal {U}}_\mathrm {P}$ with respect to 2*B*, defined by $\partial {\mathcal {U}}_\mathrm {P}/\partial B=0$, over $\varLambda$. In the bottom right inset in Fig. 6, $\varLambda _\mathrm {c}=-0.356$. We observe that ${\mathcal {U}}_{\mathcal {P}}$ depends monotonously on both *K* and *G*, and it is therefore justified for qualitative analyses to study the system for fixed values of *K* and *G* as we do in this work. If *K* or *G* are increased, for $K>0$, the critical value of the linear resistive coupling $\varLambda _\mathrm {c}$, which is computed numerically in the noise-driven case, increases in magnitude, and hence the size the transition region increases. Furthermore, for increasing *K* or *G*, the overall potential landscape becomes increasingly biased toward the synchronized solution. The top row of Fig. 6 should be interpreted with care, because at high noise levels, the amplitudes $A_j$ are in general not all simultaneously close to some amplitude $A_\beta ^*$ and the projection onto the limit cycle solutions loses its validity. Despite this, the figure illustrates correctly the monotonous trends of ${\mathcal {U}}_\mathrm {P}$, given by Eq. (28), with increasing coupling nonlinearity *K* and noise intensity *G*. The curve parametrizing the minima of ${\mathcal {U}}_\mathrm {P}$ with respect to 2*B* over $\varLambda$ is obtained by numerically computing the zero level set of $\partial {\mathcal {U}}_\mathrm {P}/\partial B$.

For $t\rightarrow \infty$, in the steady state, the probability *P* of the fast system (2) being within a domain ${\mathcal {D}}$ in the 2*N*-dimensional phase space of the fast system (2), $\varSigma =\{\eta _1,{\dot{\eta }}_1,\ldots ,\eta _N,{\dot{\eta }}_N\}$, is defined as

29 $$\begin{aligned}&P({\mathcal {D}},t\rightarrow \infty )=\nonumber \\&\int _{\mathcal {D}} {\mathcal {P}}^\infty (\eta _1,{\dot{\eta }}_1,\ldots ,\eta _N,{\dot{\eta }}_N) d\eta _1 d{\dot{\eta }}_1\ldots d\eta _N d{\dot{\eta }}_N, \end{aligned}$$

where the stationary PDF ${\mathcal {P}}^\infty ({\varvec{z}})$ of the fast variables

30 $$\begin{aligned} {\varvec{z}}=(\eta _1,{\dot{\eta }}_1,\ldots ,\eta _N,{\dot{\eta }}_N) \end{aligned}$$

can be deduced from the stationary Fokker–Planck equation (see Ref. [95]) of the slow-flow system (5) and (6), given by

31 $$\begin{aligned} 0=\frac{\partial }{\partial {\varvec{x}}}\bigg [\frac{\partial {\mathcal {V}}({\varvec{x}})}{\partial {\varvec{x}}} {\mathcal {P}}^\infty ({\varvec{x}})+\frac{\varGamma }{4\omega _0^2}\frac{\partial {\mathcal {P}}^\infty ({\varvec{x}})}{\partial {\varvec{x}}}\bigg ]^T, \end{aligned}$$

where the newly introduced (slow) variables

32 $$\begin{aligned} {\varvec{x}}=(U_1,V_1,\ldots ,U_N,V_N) \end{aligned}$$

are related to ($\eta _j$, ${\dot{\eta }}_j$) by $\eta _j=A_j\cos {\phi _j}$ and ${\dot{\eta }}_j=-\omega _0 A_j\sin {\phi _j}$, where

33 $$\begin{aligned} A_j= & {} \sqrt{U_j^2+V_j^2} \end{aligned}$$

34 $$\begin{aligned} \phi _j= & {} \arctan (V_j,U_j)+\omega _0 t. \end{aligned}$$

The derivation of ${\mathcal {P}}^\infty$ is detailed in Appendix B. The final result is

35 $$\begin{aligned}&{\mathcal {P}}^\infty (\eta _m, {\dot{\eta }}_m)= \nonumber \\&{\mathcal {N}} \exp {(-4\omega _0^2 {\mathcal {V}}(A_m(\eta _m,{\dot{\eta }}_m), \varphi _m(\eta _m,{\dot{\eta }}_m))/\varGamma )}, \qquad \end{aligned}$$

$m=1,\ldots ,N$, where $A_m=\sqrt{\eta _m^2+\left( {\dot{\eta }}_m/\omega _0\right) ^2}$, $\varphi _m=-\arctan ({\dot{\eta }}_m/\omega _0,\eta _m)-\omega _0 t$, the potential ${\mathcal {V}}$ is given by Eq. (9) and ${\mathcal {N}}$ is a normalization constant such that $P({\mathcal {D}}\rightarrow \varSigma ,t)=1$. In the figures below, the PDFs are normalized by numerical integration over the shown, finite domains.

In line with our analysis of the deterministic system in the previous section, we now project ${\mathcal {P}}^\infty$ onto the phase-locked, uniform-amplitude quasi-limit cycle solutions $\{ A_j\equiv A^*_\beta ,\varDelta _j\equiv k(j)\varDelta ^*_{\beta } \}$ to obtain the projected stationary PDF ${\mathcal {P}}^\infty _\mathrm {P}={\mathcal {N}} (\widetilde{{\mathcal {P}}}^\infty _\mathrm {P})^N$, where

36 $$\begin{aligned} \widetilde{{\mathcal {P}}}^\infty _\mathrm {P}(A^*_\beta , \varDelta ^*_\beta )= \exp {(-4\omega _0^2 {\mathcal {V}}(A^*_\beta , \varDelta ^*_\beta )/N\varGamma )}. \end{aligned}$$

This result implies that on the submanifold of phase-locked, uniform-amplitude quasi-limit cycles, all oscillators perform uncorrelated stochastically fluctuating motion around the deterministic limit cycles and that in the steady state, the PDF of each of the oscillators, up to normalization, is given by ${\widetilde{P}}^\infty _\mathrm {P}$.

**Fig. 7:**
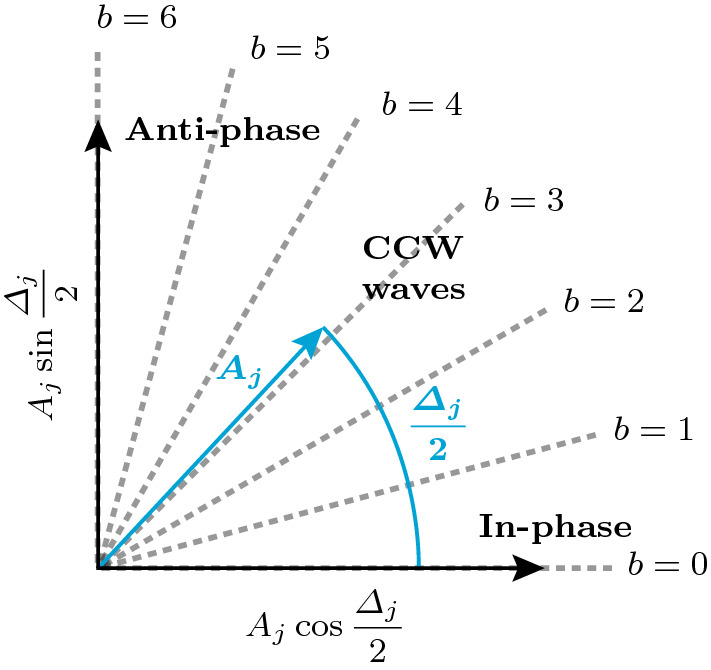
Geometric analogy between emergent patterns in the oscillator ring, quantified by the phase difference $\varDelta _j$, and polar coordinates. Possible nonnegative discrete Bloch wavenumbers $b\ge 0$ are shown for $N=12$ oscillators. The *x*-axis ($b=0$) corresponds to in-phase and the *y*-axis ($b=N/2=6$) to anti-phase synchronization. Values of *b* in between the two extremes correspond to CWW rotating waves. The lower half-plane (not shown) is symmetric, with negative values of *b* and CW waves

**Fig. 8:**
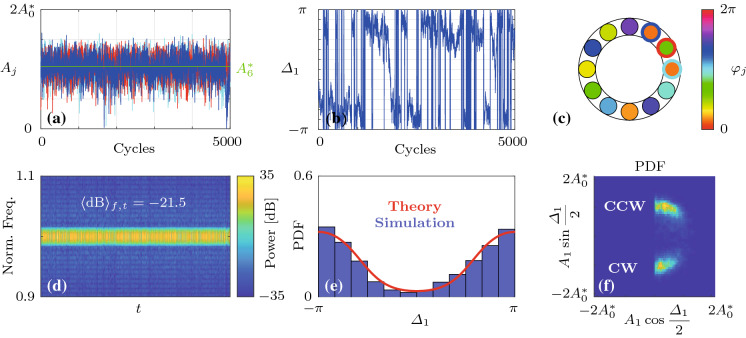
Analysis of a time trace of the slow-flow system given by Eqs. (5) and (6) with $\varLambda =-0.5$ ($\varLambda _\mathrm {c}=-0.356$) over 5000 cycles. **a** Time traces of the amplitudes $A_j$. The oscillators $j=1,2,3$ are indicated by blue, red and cyan color, respectively. The limit cycle amplitude $A_6^*$, given by Eq. (27), is shown in green. **b** Time traces of the phase difference $\varDelta _1$. Time traces of other oscillators appear similar. **c** Phase distribution $\varphi _j$ along the ring at the end time. **d** Spectrogram of the reconstructed fast oscillating signal $\eta _1(t)$, showing the estimated signal power over time *t* as a function of the normalized frequency $\omega /\omega _0$. The average of the signal power over the shown frequency and time domains, denoted by $\langle \mathrm {dB}\rangle _{f,t}$, is also indicated in the inset. **e** Comparison of the analytical PDF of the phase differences ${\widetilde{P}}^\infty _{\mathrm {P},\varDelta }$ (red) with a histogram of $\varDelta _1$ (blue) from the time trace shown in **(b)**. **f** Histograms of $A_1$ and $\varDelta _1$, represented using the geometric analogy described in Fig. 7. Spectro-and histograms of other oscillators appear similar

The PDF of the phase differences $\widetilde{{\mathcal {P}}}_{\mathrm {P},\varDelta }$ is obtained by evaluating the limit cycle amplitude $A_\beta ^*$ in Eq. (27) in terms of $\varDelta ^*_\beta$ using Eqs. (16) and (17) with $b\rightarrow \beta$ and substituting the result into the potential ${\mathcal {V}}$ in Eq. (36). The result is, up to normalization,

37 $$\begin{aligned} \widetilde{{\mathcal {P}}}_{\mathrm {P},\varDelta }^\infty (\varDelta ^*_\beta )= \exp {(-4\omega _0^2 {\mathcal {V}}(A^*_\beta (\varDelta ^*_\beta ), \varDelta ^*_\beta )/N\varGamma )}. \end{aligned}$$

To graphically compare the joint PDF $\widetilde{{\mathcal {P}}}^\infty _\mathrm {P}(A^*_\beta , \varDelta ^*_\beta )$ to normalized histograms of $A_j$ and $\varDelta _j$ obtained from time traces, we propose a geometric analogy between emergent patterns in the oscillator ring, quantified by the phase difference $\varDelta _j$, and polar coordinates. The analogy is shown in Fig. 7. Possible nonnegative discrete Bloch wavenumbers $b\ge 0$ are shown for $N=12$ oscillators. The *x*-axis ($b=0$) corresponds to in-phase and the *y*-axis ($b=N/2=6$) to anti-phase synchronization. Values of *b* in between the two extremes correspond to CWW rotating waves. The lower half-plane (not shown) is symmetric, with negative values of *b* and CW waves.

In Fig. 8, we analyze a time trace of the slow-flow system (5) and (6) with $\varLambda =-0.5$ ($\varLambda _\mathrm {c}=-0.356$), so that the considered condition lies outside the transition region. The oscillators $j=1,2,3$ are indicated by blue, red and cyan colors, respectively. In Fig. 8a, we observe that the amplitudes $A_j$ fluctuate stochastically near the deterministic limit cycle amplitude $A^*_6$, which is indicated in green. The phase difference $\varDelta _1$, shown in Fig. 8b performs similar fluctuations and jumps intermittently between $\pm \pi$. Time traces of other oscillators appear similar. The phase distribution $\varphi _j$ along the ring after 5000 cycles is shown in Fig. 8c. Figure 8d shows the spectrogram of the reconstructed fast oscillating signal $\eta _1(t)$, i.e., the estimated signal power ${\mathcal {L}}_{\eta _1 \eta _1}(f)\mathrm {\varDelta }f$, where ${\mathcal {L}}_{\eta _1 \eta _1}$ is the power spectral density of the signal $\eta _1$ and $\varDelta f$ is the equivalent noise bandwith [99] of a small frequency increment around *f* (the shown frequency domain is discretized into 257 such increments), over time *t* as a function of the normalized frequency $\omega /\omega _0$. We observe that the signal power is mainly concentrated near the natural eigenfrequency $\omega _0$. The average of the signal power over the shown frequency and time domains, denoted by $\langle \mathrm {dB}\rangle _{f,t}$, is also indicated in the inset. Figure 8e shows a comparison of the analytical PDF of the phase differences ${\widetilde{P}}^\infty _{\mathrm {P},\varDelta }$ (red) with a histogram of $\varDelta _1$ (blue) obtained from the time trace shown in Fig. 8b. As expected, for the considered conditions (strong amplifying coupling), we find symmetric bimodal distributions of the PDFs, with peaks around the anti-phase synchronized state with $\varDelta _{\pm 6}^*=\pm \pi$. Spectro- and histograms of other oscillators appear similar.

**Fig. 9:**
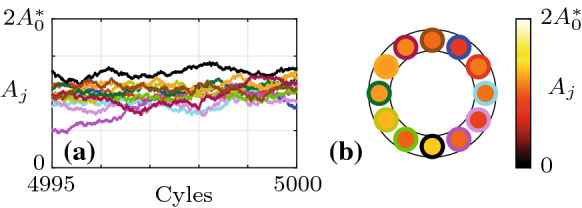
**a** Amplitudes $A_j$ of the time trace shown in Fig. 8 over the last 5 cycles. **b** Distribution of the amplitudes $A_j$ along the ring after 5000 cycles

Figure 9a depicts the amplitudes of the time trace shown in Fig. 8a over the last five cycles for $j=1,\ldots ,N$. In Fig. 9b, the distribution of the amplitudes $A_j$ along the ring at the end time is shown. We observe that the assumption of slowly varying, uniform amplitudes is approximately satisfied.

**Fig. 10:**
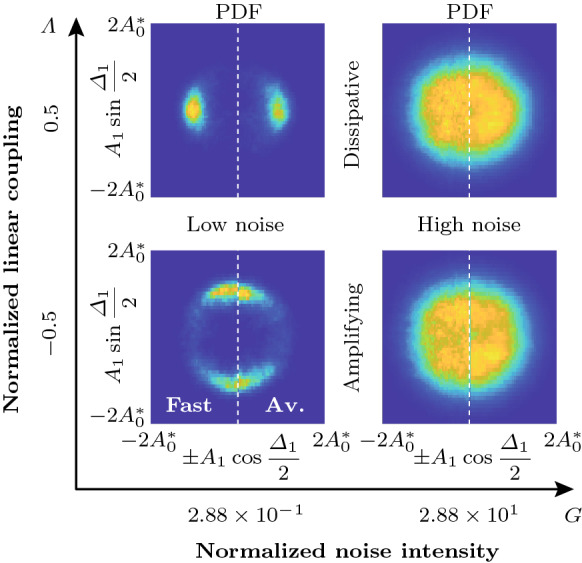
Validation of the stochastic averaging method against numerical simulations. Histograms of $A_1$ and $\varDelta _1$, computed over 10,000 cycles from the fast and the averaged system, given by Eq. (2) and Eqs. (5) and (6), respectively, are compared for varying linear resistive coupling $\varLambda$ and noise intensity *G*. Histograms of other oscillators appear similar. Note that the amplitude $A_0^*$ depends on the noise intensity *G* and changes from the left to the right column

The stochastic averaging method is validated against numerical simulations in Fig. 10 by comparing histograms of $A_1$ and $\varDelta _1$, computed over 10,000 cycles from the fast and the averaged system, given by Eq. (2) and Eqs. (5) and (6), respectively, for varying linear resistive coupling $\varLambda$ and noise intensity *G*. Histograms of other oscillators appear similar. The results show good qualitative agreement between the two methods. Both methods describe a symmetric, bimodal distribution for $\varLambda <0$. At high noise intensity, the PDF becomes more spread out and less discernible patterns are visible. Note that the amplitude $A_0^*$ depends on the noise intensity *G* and changes from the left to the right column. Sharper shapes of the PDFs emerge for longer times, but the computational cost of simulating the averaged system (5) and (6) became prohibitive for longer time traces than those shown in Fig. 10. Nevertheless, the analytical results derived above in this section enable further validation of the stochastic averaging method.

**Fig. 11:**
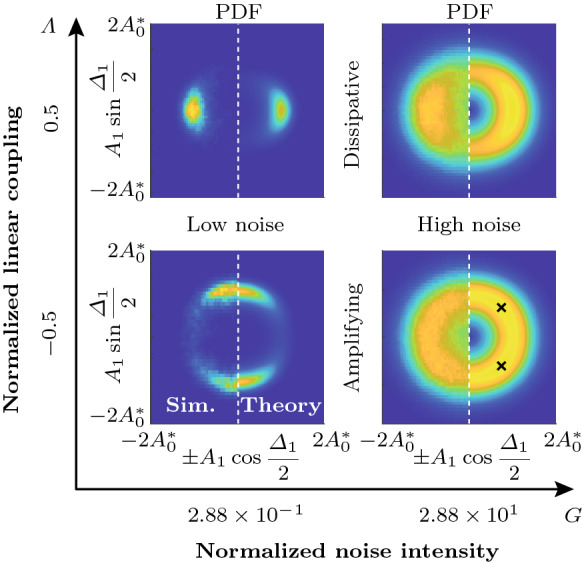
Validation of the stochastic averaging method against analytical results. Histograms of $A_1$ and $\varDelta _1$, computed over 10,000 cycles at low noise and 100,000 cycles at high noise from the fast system (2) (**Sim.**) are compared to the joint PDF $\widetilde{{\mathcal {P}}}_\mathrm {P}^\infty$ defined in Eq. (36) (**Theory**) for varying linear resistive coupling $\varLambda$ and noise intensity *G*. For high noise and amplifying coupling, the maxima of the PDF are indicated by black crosses. Histograms of the other oscillators appear similar. Note that the amplitude $A_0^*$ changes from the left to the right column

**Fig. 12:**
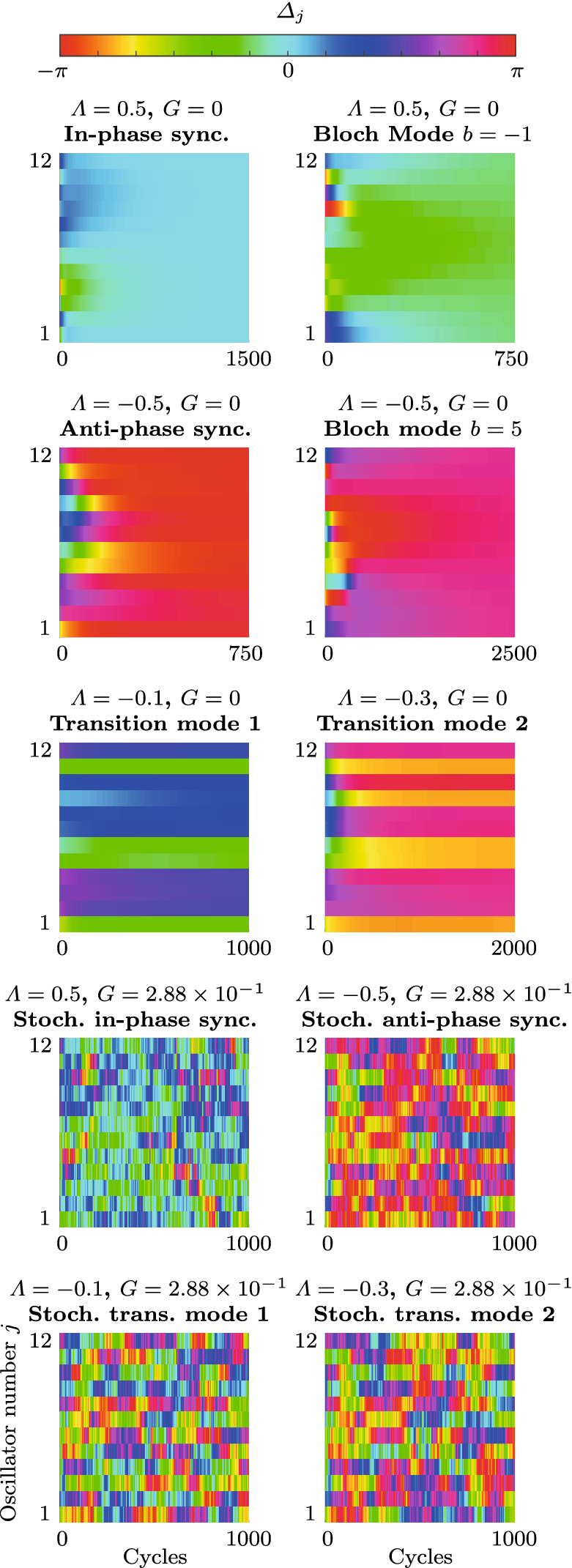
Transients of deterministic time traces contained in the Supplementary Material, showing the color-coded phase differences $\varDelta _j$ as a function of time and the oscillator number *j*. For the stochastic time traces, the figure depicts the evolution over the first 1000 cycles. Parameter values are indicated over the respective insets

In Fig. 11, the stochastic averaging method is validated against analytical results by comparing histograms of $A_1$ and $\varDelta _1$ computed from the fast system (2) (**Sim.**) to the joint PDF $\widetilde{{\mathcal {P}}}_\mathrm {P}^\infty$, given by Eq. (36) (**Theory**), for varying linear resistive coupling $\varLambda$ and noise intensity *G*. Histograms of other oscillators appear similar. At low noise, time traces were computed for 10,000 cycles and at high noise for 100,000 cycles. For high noise and amplifying coupling, the maxima of the PDF are indicated by black crosses. The results show good qualitative agreement between the two methods. Note that the amplitude $A_0^*$ depends on the noise intensity *G* and changes from the left to the right column.

**Fig. 13:**
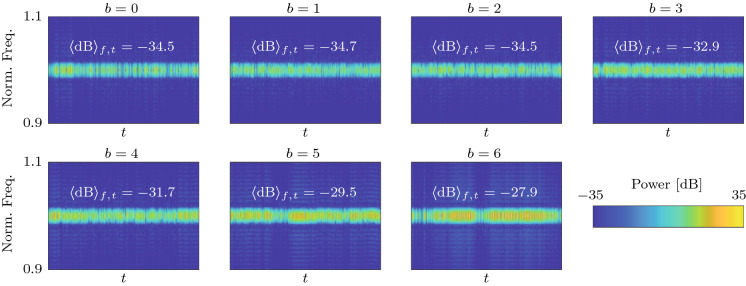
Spectrograms of the modal amplitudes $\eta _{b}$ for $b=0,\ldots ,6$, showing the estimated signal power as a function of time *t* and the normalized frequency $\omega /\omega _0$. The modal amplitudes $\eta _{b}$ are obtained by decomposing the reconstructed fast pressure signals $\eta _j$ from the example shown in Fig. 8 (time trace of the slow-flow system (5) and (6) over 5000 cycles for $\varLambda =-0.5$) into different Bloch mode components using the discrete Fourier transform, see Eq. (38). Spectrograms for positive and negative *b* appear similar. The average of the signal power over the shown frequency and time domains, denoted by $\langle \mathrm {dB}\rangle _{f,t}$, is also indicated in the insets

For completeness, we show in Fig. 12 transients of the deterministic time traces contained in the Supplementary Material, showing the color-coded phase differences $\varDelta _j$ as a function of time and the oscillator number *j*. For the stochastic time traces, the figure depicts the evolution over the first 1000 cycles. Parameter values are indicated over the respective insets.

**Fig. 14:**
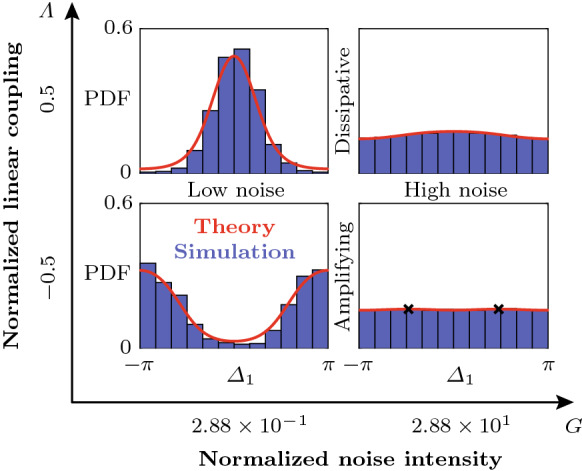
Comparison of the stationary PDF of the phase differences $\widetilde{{\mathcal {P}}}^\infty _{\mathrm {P},\varDelta }(\varDelta _b^*)$, given by Eq. (37) (**Theory**), to histograms of $\varDelta _1$ obtained from time series simulations of the fast system (2) over 15,000 cycles (**Simulation**). For high noise and amplifying coupling, the maxima of the PDF are indicated by black crosses. Histograms obtained from other oscillators or from the averaged system (5) and (6) appear similar

The spectrograms of $\eta _j$, shown in Fig. 8d for $j=1$, can be decomposed into spectrograms of different rotating wave components by defining the modal amplitude $\eta _{b}$ of the Bloch mode with wavenumber *b* using the discrete Fourier transform [25]:

38 $$\begin{aligned} \eta _b(t)=\frac{1}{N}\sum ^N_{k=1} \eta _k(t) e^{-i b 2\pi k /N}. \end{aligned}$$

In Fig. 13, we plot spectrograms of $\eta _b$ for $b=0,\ldots ,6$. Spectrograms for positive and negative *b* appear similar. Random, intermittent energy transfer between different modes is observed. The average of the signal power over the shown frequency and time domains, denoted by $\langle \mathrm {dB}\rangle _{f,t}$, is also indicated in the insets. As expected, for the given conditions ($\varLambda =-0.5$, $\varLambda _\mathrm {c}=-0.356$), the anti-phase synchronized state with $b=\pm 6$ dominates the power spectrum.

In Fig. 14, the stationary PDF of the phase differences $\widetilde{{\mathcal {P}}}^\infty _{\mathrm {P},\varDelta }$, given by Eq. (37), is compared to histograms of $\varDelta _1$ obtained from time series simulations of the fast system (2) over 15,000 cycles for varying noise intensity *G* and coupling nonlinearity *K*. For high noise and amplifying coupling, the maxima of the PDF are indicated by black crosses. The plots show good agreement between the two methods. Histograms obtained from other oscillators or from time traces of the averaged system (5) and (6) appear similar.

**Fig. 15:**
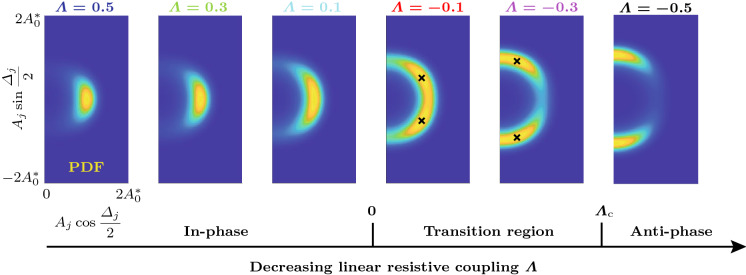
Visualization of the (de-)synchronization transition. Shown is the evolution of the joint PDF $\widetilde{{\mathcal {P}}}^\infty _\mathrm {P}$, given by Eq. (36), for varying $\varLambda$ with $G=2.88\times 10^{-1}$ (“Low noise” in Fig. 11). The values of the normalized linear resistive coupling $\varLambda$ correspond to those used in the numerical experiments reported in Fig. 4. In the transition region, the maxima of the PDF are indicated by black crosses. For the given conditions, $\varLambda _\mathrm {c}=-0.356$

The evolution of the joint PDF $\widetilde{{\mathcal {P}}}^\infty _\mathrm {P}$, given by Eq. (36), is visualized in Fig. 15 for varying $\varLambda$ ($\varLambda _\mathrm {c}=-0.356$), with $G=2.88\times 10^{-1}$ (“Low noise” in Fig. 11). The values of the normalized linear resistive coupling $\varLambda$ correspond to those used in the numerical experiments reported in Fig. 4. In the transition region, the maxima of the PDF are indicated by black crosses. The figure demonstrates how the above analysis, combined with the geometric analogy introduced in Fig. 7, enables a compact description of the (de-)synchronization transition in dependence of $\varLambda$, independent of the number of oscillators *N*. The joint PDF $\widetilde{{\mathcal {P}}}^\infty _\mathrm {P}$ reproduces the transition from a unimodal to a bimodal distribution as $\varLambda$ is varied from 0.5 to $-0.5$, which was also observed in the numerical experiments on the deterministic system reported in Figs. 3 and 4.

In this section, we analyzed the steady-state statistics of the noise-driven ring of oscillators. We introduced a geometric analogy between the emergent patterns in the ring, quantified by the phase difference $\varDelta _j$, and polar coordinates to enable a graphical description of the (de-)synchronization transition as a function of the linear resistive coupling. The stationary joint PDF $\widetilde{{\mathcal {P}}}^\infty _\mathrm {P}$ and the PDF of the phase differences $\widetilde{{\mathcal {P}}}^\infty _{\mathrm {P},\varDelta }$ were obtained by projecting the exact solution of the stationary Fokker–Planck equation onto the phase-locked, uniform-amplitude quasi-limit cycle solutions. The stochastic averaging method was validated by comparing histograms from time series of the fast to those of the averaged system and to the analytical PDFs, showing good agreement over the parameter range considered.

**Fig. 16:**
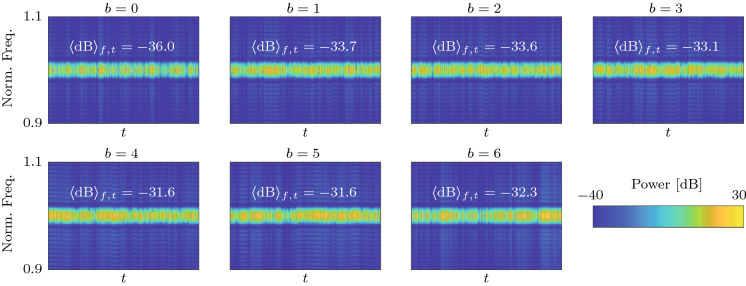
Spectrograms of the modal amplitudes $\eta _{b}$ for $b=0,\ldots ,6$, showing the estimated signal power as a function of time *t* and the normalized frequency $\omega /\omega _0$. The modal amplitudes $\eta _{b}$ are obtained by decomposing the pressure signals $\eta _j$ from time series of the fast system (2) with $\varLambda =-0.3$ ($\varLambda _\mathrm {c}=-0.356$) over 5000 cycles into different Bloch mode components using the discrete Fourier transform, see Eq. (38). Spectrograms for positive and negative *b* appear similar. The average of the signal power over the shown frequency and time domains, denoted by $\langle \mathrm {dB}\rangle _{f,t}$, is also indicated in the insets

## Discussion

In Fig. 13, spectrograms of the modal amplitudes $\eta _b$ of Bloch modes with different wavenumber *b* were shown. In Fig. 8 of Ref. [25], similar spectrograms were obtained from acoustic pressure measurements at the can outlets ($\sim \eta _j$) of a real-world gas turbine with $N=12$ cans. The difference is that in the former, the Bloch modes with $b=5,6$ dominate the power spectrum, while in the latter, the modes with $b=3,4$ are dominant. By decreasing $\varLambda$ to values within the transition region, this clustering of the signal power around non-maximal Bloch wavenumbers *b* can be reproduced. The corresponding spectrograms are shown in Fig. 16, showing the distribution of the estimated signal power over time *t* as a function of the normalized frequency $\omega /\omega _0$. The time traces were computed for $\varLambda =-0.3$ ($\varLambda _\mathrm {c}=-0.356$) with the fast system (2). Spectrograms for positive and negative *b* appear similar. The average of the signal power over the shown frequency and time domains, denoted by $\langle \mathrm {dB}\rangle _{f,t}$, is also indicated in the insets. We observe that the signal power is now concentrated around the Bloch modes with $b=4,5$. If $\varLambda$ is further decreased, the signal power shifts toward lower values of *b*. Note that the inclusion of a coupling nonlinearity *K* in the model is necessary to observe this effect, as without it, there is no transition region (see Fig. 6). The intermittent energy transfer between different Bloch waves observed in Ref. [25] is qualitatively reproduced by our model. It is an interesting insight that despite the signal power appearing in the spectrograms of the Bloch modes with discrete wavenumber *b*, the underlying deterministic dynamics are mainly dominated by quasi-steady, non-classical transition modes which are superpositions of CW and CCW rotating waves. This fact cannot be understood from simple observation of the spectrograms, which may suggest that the Bloch modes exclusively dominate the underlying dynamics. The spectrograms reported in Ref. [25] show a shift of the frequency around which the signal power is concentrated with varying Bloch wavenumber *b*. This feature is not reproduced by our model, which may be because the reactive coupling was neglected. Besides including reactive coupling effects, a topic for future research is to quantify in more detail the time-dependent energy transfer between different Bloch waves observed in both real-world experiments and our model. For such transient phenomena, which are not the focus of this study, differences between the fast system (2) and the averaged system (5) and (6) are expected in the presence of noise, i.e., for $\varGamma \ne 0$.

We note that several questions remain open regarding the transition modes observed in Sect. 3 including, but not limited to, the following:

- Are there different types of transition modes, distinguished by the distribution of $\varDelta _j$ along the ring, and can they be systematically classified?
- How is the ring size *N* related to the complexity of the transition modes?
- What is their sensitivity with respect to initial conditions? Especially, is it possible to predict, from the initial conditions, if a trajectory will converge to a discrete Bloch mode or to a transition mode?

The plots in Fig. 14 show that at low noise, the PDF of the phase differences is highly sensitive with respect to changes in $\varLambda$. Another topic for future research could be to investigate whether this sensitivity, described by the analytical expression for ${\widetilde{P}}^\infty _{\mathrm {P},\varDelta }$ given in Eq. (37), can be exploited to perform parameter identification of the linear resistive coupling $\lambda$.

The following are also topics for future research: Asymmetries have been neglected in this study, but are inherent in real systems. To understand their effects, further investigation is required. Furthermore, in real gas turbines, “true” azimuthal waves may appear in the continuous, annular plenum concurrently with can-annular modes, i.e., apparent rotating waves that emerge due to communicating thermoacoustic modes in individual, but coupled control volumes. The classification of these combined modes is also a topic for future research.

The results reported in Figs. 3 and 4 bear some similarity to the studies found in Refs. [33, 67, 100] where phase-flip bifurcations are studied. The difference is that the referenced works consider transient bifurcations which occur during operation as some parameter is varied, while our investigation is focused on steady-state phenomena with all parameters constant during operation. Extending the current approach to the non-stationary case, and studying transient bifurcations in a similar system could prove a fruitful direction for subsequent studies.

**Fig. 17:**
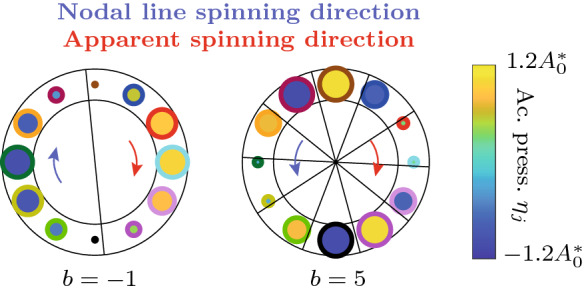
Spatial analogue of the wagon-wheel effect. The acoustic pressure $\eta _j$ along the ring is visualized at a fixed time *t* for $b=-1$ and $b=5$, respectively. The diametric black lines indicate the nodal lines. The radii of the small circles scale with $|\eta _j|$. As the nodal lines of the $b=5$ mode spin in CCW direction, they first hit the oscillators with $j=3,9$ and then those with $j=4,10$ etc. Thus, there appears to be only a single nodal line which spins in CW direction, although the underlying Bloch mode is a CCW wave with five nodal lines. Movies of the corresponding time traces are included in the Supplementary Material

We conclude our discussion with the following remarks: As described in Sect. 3, Bloch modes with positive or negative wavenumber *b* appear as CW or CCW rotating waves, i.e., waves whose nodal lines spin in CW or CCW direction. A Bloch mode with wavenumber *b* has exactly |*b*| nodal lines. However, for finite *N*, if $\mathrm {mod}(N,|b|)\ne 0$, there appears to be only a single nodal line which spins in the opposite direction. This can be understood as a spatial analogue of the wagon-wheel effect, where wheels appear to spin in the opposite direction as an effect of finite temporal sampling rates [97]. The effect is illustrated in Fig. 17, where the acoustic pressure $\eta _j$ along the ring is shown at a fixed time *t*. The diametric black lines indicate the nodal lines. The radii of the small circles scale with $|\eta _j|$. As the nodal lines of the $b=5$ mode spin in CCW direction, they first hit the oscillators with $j=3,9$ and then those with $j=4,10$ etc. Thus, there appears to be only a single nodal line which spins in CW direction, although the underlying Bloch mode is a CCW wave with five nodal lines. Movies of the corresponding time traces are included in the Supplementary Material.

## Conclusions

In this work, a ring of noise-driven oscillators was analyzed. Deterministic and stochastic averaging was performed to eliminate the fast oscillating terms. Numerical experiments were performed on the noise-free system to motivate the direction of the study. By projecting the potential of the slow-flow variables onto the phase-locked, uniform-amplitude quasi-limit cycle solutions, a compact description of the (de-)synchronization transition in the ring was obtained. These results were adapted to the noise-driven ring of oscillators to derive, in analytical form, the steady-state statistics of the fast system. Special focus was placed on the phase difference $\varDelta _j$, which quantifies emergent patterns along the ring. The stochastic averaging procedure was validated against numerical simulations and analytical results. We have demonstrated that a simple oscillator model with symmetric, purely resistive coupling can reproduce the intermittent energy transfer between different Bloch mode components observed in real-world gas turbines.

## Data Availability

The datasets used for generating the plots and results in the present study can be directly obtained from the numerical simulation of the related mathematical equations in the manuscript.

## Appendix

### Deterministic and stochastic averaging

Below, we perform deterministic and stochastic averaging on the fast system (2) to obtain the slow-flow equations (5) and (6) for the amplitudes $A_j$ and the phases $\varphi _j$, respectively. These variables describe the dynamics of the envelope of the acoustic pressure oscillations in the cans. We follow closely the derivation presented in Ref. [70] for a single Van der Pol oscillator.

Considering the averaged system as an approximation for the amplitude dynamics in fast system is justified when **(A)** the fast time scale ($\sim 1/\omega _0$) is clearly separated from the slow time scale of the amplitude and phase variation ($\sim 1/\nu$), and **(B)** the fast system corresponds to weakly perturbed harmonic oscillators performing quasi-sinusoidal motion (see [91, 92] for deterministic and [93, 94] for stochastic averaging). Thermoacoustic interactions in combustion chambers typically fall into the category of weakly damped/driven oscillations with $|\nu |\ll \omega _0$. Furthermore, the aeroacoustic coupling is assumed to be weak, i.e., $|\lambda |\ll \nu$, so that assumption **(A)** is generally satisfied. Furthermore, under those assumptions, the nonlinearity will also be small because at full saturation, when the limit cycle is reached, in the absence of coupling, $\eta _j\sim \sqrt{\nu /\kappa }$ so that $\kappa \eta _j^2\sim \nu$. As shown in Sect. 3, under the present assumption of weak coupling ($|\varLambda |\ll 1$, $K\ll 1$), the limit cycle amplitude is not significantly affected by the resistive coupling terms. Therefore, the RHS of Eq. (2) is generally small compared to the LHS, and assumption **(B)** is satisfied.

We limit ourselves to the case of perfectly tuned oscillators with identical eigenfrequencies $\omega _0$. To derive the slow-flow dynamics, we first make a coordinate change to amplitude-phase variables $\{A_j,\phi _j\}$, $j=1,\ldots ,N$:

39 $$\begin{aligned} A_j= & {} \sqrt{\eta _j^2+\left( {\dot{\eta }}_j/\omega _0\right) ^2}, \end{aligned}$$

40 $$\begin{aligned} \varphi _j= & {} -\arctan ({\dot{\eta }}_j/\omega _0,\eta _j)-\omega _0t, \end{aligned}$$

where $\eta _j=A_j\cos {\phi _j }$, ${\dot{\eta }}_j=-\omega _0A_j\sin {\phi _j }$, $\phi _j=\omega _0t+\varphi _j$ and $\arctan (y,x)$ is the two-argument arctangent function. To transform Eq. (2), we rewrite it as

41 $$\begin{aligned} \ddot{\eta }_{j}+\omega _0 ^2{\eta }_j =f(\eta _k,{\dot{\eta }}_k)+\xi _j, \end{aligned}$$

for $$k=j-1,j,j+1$$, where

42 $$\begin{aligned}&f(\eta _k,{\dot{\eta }}_k)=2\nu {\dot{\eta }}_j-\kappa \eta _j^2{\dot{\eta }}_j+ \lambda ({\dot{\eta }}_{j+1}+{\dot{\eta }}_{j-1}-2{\dot{\eta }}_j)\nonumber \\&\quad +\vartheta \big [({\eta }_{j+1}-{\eta }_j)^2({\dot{\eta }}_{j+1}-{\dot{\eta }}_j)+({\eta }_{j-1}-{\eta }_j)^2({\dot{\eta }}_{j-1}\nonumber \\&\quad -{\dot{\eta }}_j)\big ]. \end{aligned}$$

Taking the time derivative of Eqs. (39) and (40) and substituting Eq. (41) into the resulting expressions yields

43 $$\begin{aligned} {\dot{A}}_j= & {} f_1(A_k,\phi _k)+f_2(A_k,\phi _k), \end{aligned}$$

44 $$\begin{aligned} {\dot{\varphi }}_j= & {} f_3(A_k,\phi _k)+f_4(A_k,\phi _k), \end{aligned}$$

where

45 $$\begin{aligned} f_1(A_k,\phi _k)= & {} -\frac{\sin {\phi _j}}{\omega _0}f(A_k\cos {\phi _k },-\omega _{0}A_k\sin {\phi _k }), \end{aligned}$$

46 $$\begin{aligned} f_2(A_k,\phi _k)= & {} -\frac{\sin {\phi _j}}{\omega _0}\xi _j, \end{aligned}$$

47 $$\begin{aligned} f_3(A_k,\phi _k)= & {} -\frac{\cos {\phi _j}}{\omega _0 A_j}f(A_k\cos {\phi _k },-\omega _{0}A_k\sin {\phi _k }), \end{aligned}$$

48 $$\begin{aligned} f_4(A_k,\phi _k)= & {} -\frac{\cos {\phi _j}}{\omega _0 A_j}\xi _j, \end{aligned}$$

for $k=j-1,j,j+1$. The expressions in Eqs. (45) and (47) are valid for any nonlinear function *f*. We now restrict ourselves the case where the function *f* is given by Eq. (42). Averaging is applied to keep only contributions that are relevant on the slow time scale associated with the modulation of the amplitudes $A_j$ and phases $\psi _j$. First, following the classic treatment of Krylov and Bogoliubov [91], deterministic averaging is performed on $f_1$ and $f_3$. To facilitate the averaging method, we rewrite these functions by expanding the trigonometric terms. This yields

49 $$\begin{aligned}&f_1(A_k,\phi _k)=A_j\bigg (\nu -\frac{\kappa A_j^2}{8}\bigg )+\frac{\lambda }{2}\big [A_{j+1}\cos {(\varDelta _{j+1})}\nonumber \\&\quad +A_{j-1}\cos {(\varDelta _{j})}-2A_j\big ]+\frac{\vartheta }{8}\Big [(A_{j+1}^3+3A_j^2 A_{j+1})\nonumber \\&\quad \cos (\varDelta _{j+1})-A_j A_{j+1}^2\cos (2\varDelta _{j+1})+(A_{j-1}^3\nonumber \\&\quad +3A_j^2 A_{j-1})\cos (\varDelta _{j-1})-A_j A_{j-1}^2\cos (2\varDelta _{j-1})\nonumber \\&\quad -2(A_j^3+A_j A_{j+1}^2+A_j A_{j-1}^2)\Big ]+\text {f.o.t.} \end{aligned}$$

and

50 $$\begin{aligned}&f_3(A_k,\phi _k)=\frac{\lambda }{2}\left[ \frac{A_{j+1}}{A_j}\sin (\varDelta _{j+1})-\frac{A_{j-1}}{A_j}\sin (\varDelta _{j})\right] \nonumber \\&\quad +\frac{\vartheta }{8}\Bigg [\bigg (A_j A_{j+1}+\frac{A_{j+1}^3}{A_j}\bigg )\sin (\varDelta _{j+1})-A_{j+1}^2\nonumber \\&\quad \sin (2\varDelta _{j+1})-\bigg (A_j A_{j-1}+\frac{A_{j-1}^3}{A_j}\bigg )\sin (\varDelta _{j-1})\nonumber \\&\quad +A_{j-1}^2\sin (2\varDelta _{j-1})\Bigg ]+\text {f.o.t.} \end{aligned}$$

where $\varDelta _j=\phi _j-\phi _{j-1}=\varphi _j-\varphi _{j-1}$ and “f.o.t.” stands for “fast oscillating terms.” Note that the quantities $\phi _j+\phi _{j\pm 1}=\varphi _j+\varphi _{j\pm 1}+2\omega _0 t$ as well as $2\phi _j=2\varphi _j+2\omega _0 t$ define arguments of fast oscillating terms. We assume that the period of an acoustic cycle $T=2\pi /\omega _0$ is small compared to the slow time scale $1/\nu$ of the modulation of the amplitude and phase signals. By taking the averages of Eqs. (49) and (50), $\langle f_1(A_k,\varphi _k)\rangle _T$ and $\langle f_3(A_k,\varphi _k)\rangle _T$, respectively, over a time interval $\{t_0, t_0+T\}$ of length *T* with arbitrary start time $t_0$, one removes the fast oscillating terms.

Stochastic averaging of the terms $f_2$ and $f_4$ can be performed in different ways, two of which are presented in Refs. [93] (Vol. 2, pp. 105-113) and [94], respectively. The former method, due to Stratonovich, is adopted here. In our derivation, we follow the presentation of a similar result (stochastic averaging of a single thermoacoustic van der Pol oscillator) given in Appendix A of Ref. [70].

For compactness, we use the notation $f_\tau =f(t+\tau )$ and the dependence of $\phi _j$, $\varphi _j$ and $A_j$ on time is suppressed below. We introduce the random variables $\sigma _{j,1}=-\xi _j\sin {\phi _j}=f_2\omega _0$ and $\sigma _{j,2}=-\xi _j\cos {\phi _j}=f_4 A_j \omega _0$. We begin by estimating the expected values of $\sigma _{j,1}$ and $\sigma _{j,2}$. We consider a time interval of length $\theta$ satisfying $1/\nu \gg \theta \gg \mathrm {max}(\tau _{\xi _j},\tau _{A_j},\tau _{\varphi _j})\quad \forall j$, where $\tau _{a}$ denotes the correlation time of the signal “*a*”. We note that although the signals $\xi _j$, $\zeta _j$ and $\chi _j$ are modeled as white noise sources with (theoretically) vanishing correlation times, in reality and in numerical simulations, these signal will generally have nonzero, albeit small correlation times. As a first step, we approximate $\sigma _{j,1}$ and $\sigma _{j,2}$ as follows:

51 $$\begin{aligned}&\sigma _{j,1}=-\xi _j(t)\sin {(\omega _0 t+\varphi _j)} \nonumber \\&\quad \approx -\xi _j \sin {(\omega _0 t+\varphi _{j,-\theta })}-\xi _j\cos {(\omega _0 t + \varphi _{j,-\theta })}\mathrm {\varDelta } \varphi _j, \nonumber \\ \end{aligned}$$

52 $$\begin{aligned}&\sigma _{j,2}=-\xi _j(t)\cos {(\omega _0 t+\varphi _j)}\nonumber \\&\quad \approx -\xi _j \cos {(\omega _0 t+\varphi _{j,-\theta })}+\xi _j\sin {(\omega _0 t + \varphi _{j,-\theta })}\mathrm {\varDelta } \varphi _j,\nonumber \\ \end{aligned}$$

where $\varphi _{j,-\theta }$=$\varphi _j(t-\theta )$ and $\mathrm {\varDelta } \varphi _j=\varphi _j-\varphi _{j,-\theta }$. Using the fact that $\varphi _{j,-\theta }$ is not correlated with $\xi _j$ since $\theta \gg \mathrm {max}(\tau _{\xi _j},\tau _{\varphi _j})$, we write

53 $$\begin{aligned}&\langle \sigma _{j,1}\rangle _{\mathbb {R}} \approx -\cos { \phi _j}\langle \xi _j \mathrm {\varDelta } \varphi _j \rangle _{\mathbb {R}} \end{aligned}$$

54 $$\begin{aligned}&\langle \sigma _{j,2}\rangle _{\mathbb {R}} \approx \sin { \phi _j}\langle \xi _j \mathrm {\varDelta } \varphi _j \rangle _{\mathbb {R}}, \end{aligned}$$

where $\langle \cdot \rangle _{\mathbb {R}}$ denotes the time integral over the real line $[-\infty ,\infty ]$. Next, we integrate Eq. (44) over the time interval $[t-\theta ,t]$. Integrating Eq. (48) without the fast oscillation terms, which vanish after averaging over one period (see Eq. (50)), we can write

55 $$\begin{aligned}&\mathrm {\varDelta }\varphi _j=\int ^t_{t-\theta } -\frac{\xi _j}{ \omega _0 A_j} \cos {\varphi _j} {d}t \nonumber \\&\quad =\int ^0_{-\theta }-\frac{\xi _j(\tau +t)}{\omega _0 A_j(\tau +t)}\cos \big (\omega _0(\tau +t)+\varphi _j(\tau +t)\big ) d\tau .\nonumber \\ \end{aligned}$$

Considering that $\xi _j$ is a stationary process and that $1/\nu \gg \theta$, i.e., the variation of the amplitudes $A_j$ and phases $\varphi _j$ over the considered time interval is negligible, we obtain

56 $$\begin{aligned}&\langle \xi _j \mathrm {\varDelta } \varphi _j\rangle _{\mathbb {R}}=\nonumber \\&\quad -\frac{1}{\omega _0 A_j} \int ^0_{-\theta } \langle \xi _j \xi _{j,\tau }\rangle _{\mathbb {R}} \cos {(\omega _0(\tau +t)+\varphi _j)} d\tau \nonumber \\ \end{aligned}$$

and

57 $$\begin{aligned}&\langle \sigma _{j,1}\rangle _{\mathbb {R}} \nonumber \\&\quad =\frac{\int ^0_{-\theta } \langle \xi _j \xi _{j,\tau } \rangle _{\mathbb {R}} \cos {(\omega _0 t +\varphi _j)}\cos {(\omega _0 (\tau +t)+\varphi _j)} d\tau }{\omega _0 A_j}, \nonumber \\ \end{aligned}$$

58 $$\begin{aligned}&\langle \sigma _{j,2}\rangle _{\mathbb {R}} \nonumber \\&\quad = -\frac{\int ^0_{-\theta } \langle \xi _j \xi _{j,\tau } \rangle _{\mathbb {R}} \sin {(\omega _0 t +\varphi _j)}\cos {(\omega _0 (\tau +t)+\varphi _j)} d\tau }{\omega _0 A_j}. \nonumber \\ \end{aligned}$$

Expanding the products of cosines in Eqs. (57) and (58) and neglecting the fast oscillating terms gives

59 $$\begin{aligned} \langle \sigma _{j,1}\rangle _{\mathbb {R}}= & {} \frac{\int ^0_{-\infty } \langle \xi _j \xi _{j,\tau } \rangle _{\mathbb {R}} \cos {(\omega _0 \tau +\varphi _j)} d\tau }{2\omega _0 A_j}\nonumber \\= & {} \frac{\pi {\mathcal {L}}_{\xi _j \xi _j}(\omega _0)}{2\omega _0 A_j}=\frac{\varGamma }{4\omega _0 A_j} \end{aligned}$$

60 $$\begin{aligned} \langle \sigma _{j,2}\rangle _{\mathbb {R}}= & {} -\frac{\int ^0_{-\infty } \langle \xi _j \xi _{j,\tau } \rangle _{\mathbb {R}} \sin {(\omega _0 t)} d\tau }{\omega _0 A_j}\approx 0, \end{aligned}$$

where ${\mathcal {L}}_{\xi _j \xi _j}$ is the power spectral density of the noise signal $\xi _j$.

We proceed by estimating the correlation function of the zero mean processes $\sigma _{j,1}'=\sigma _{j,1}-\langle \sigma _{j,1}\rangle _{\mathbb {R}}$ and $\sigma _{j,2}'=\sigma _{j,2}-\langle \sigma _{j,2}\rangle _{\mathbb {R}}=\sigma _{j,2}$. In Eqs. (51) and (52), the mean values are determined by the second terms on the RHS and the fluctuating components result from the first terms:

61 $$\begin{aligned} \sigma _{j,1}'\approx & {} -\xi _j \sin {(\omega _0 t+\varphi _{j,-\theta })} \end{aligned}$$

62 $$\begin{aligned} \sigma _{j,2}'\approx & {} -\xi _j \cos {(\omega _0 t+\varphi _{j,-\theta })}. \end{aligned}$$

Since $\theta \gg \mathrm {max}(\tau _{\xi _j},\tau _\varphi )$, it is assumed that these processes are delta-correlated. Considering first $\sigma _{j,1}'$, one can write

63 $$\begin{aligned} \langle \sigma _{j,1}' \sigma _{j,1,\tau }'\rangle _{\mathbb {R}}=\delta (\tau )\int ^\infty _{-\infty } \langle \sigma _{j,1}' \sigma _{j,1,\varTheta }'\rangle d\varTheta , \end{aligned}$$

where the integral in Eq. (63) is expressed as

$$\begin{aligned}&\int ^\infty _{-\infty }\langle \xi _j \sin {(\omega _0 t+\varphi _{j,-\theta })}\xi _{j,\varTheta }\\&\quad \times \sin {(\omega _0 (t+\varTheta )+\varphi _{j,-\theta })}\rangle _{\mathbb {R}} d\varTheta . \end{aligned}$$

Because the correlation time $\tau _{\xi _j}$ is much shorter (theoretically zero) than the oscillation period $1/\omega _0$, we can neglect the fast oscillating terms and write

64 $$\begin{aligned} \langle \sigma _{j,1}' \sigma _{j,1,\tau }'\rangle _{\mathbb {R}}= & {} \delta (\tau ) \int ^\infty _{-\infty } \langle \xi _j \xi _{j,\varTheta } \rangle _{\mathbb {R}} \frac{\cos (\omega _0 \varTheta )}{2}d\varTheta \nonumber \\= & {} \pi {\mathcal {L}}_{\xi _j \xi _j}(\omega _0)\delta (\tau ) \nonumber \\= & {} \frac{\varGamma }{2}\delta (\tau ). \end{aligned}$$

Following the same derivation for $\sigma _{j,2}'$ yields the correlation function $\langle \sigma _{j,2}' \sigma _{j,2,\tau }'\rangle _{\mathbb {R}}=(\varGamma /2)\delta (\tau )$. With the above results, we can write

65 $$\begin{aligned} \langle f_2(A_k,\varphi _k)\rangle _{\mathbb {R}}= & {} \frac{\varGamma }{4\omega _0^2 A_j}+\zeta _j, \end{aligned}$$

66 $$\begin{aligned} \langle f_4(A_k,\varphi _k)\rangle _{\mathbb {R}}= & {} \frac{\chi _j}{A_j}, \end{aligned}$$

for $j=1,\ldots ,N$, where $\zeta _j$ and $\chi _j$ are 2*N* uncorrelated white noise sources with the noise intensity $\varGamma /2\omega _0^2$. Note that the mean of the averaged process $\langle f_2(A,\phi )\rangle _{\mathbb {R}}$ is not zero and that the stochastic averaging procedure gives rise to a deterministic term which is inversely proportional to $A_j$.

Combining Eqs. (49), (50), (65) and (66), together with the assumption that the variation of $A_j$ and $\varphi _j$ over an acoustic period is negligible, yields the slow-flow amplitude and phase dynamics of the fast system (2), given by the following set of equations:

67 $$\begin{aligned}&{\dot{A}}_j=A_j\bigg (\nu -\frac{\kappa A_j^2}{8}\bigg )+\frac{\lambda }{2}\big [A_{j+1}\cos {(\varDelta _{j+1})}+A_{j-1}\nonumber \\&\quad \cos {(\varDelta _{j})}-2A_j\big ]+\frac{\vartheta }{8}\bigg [(A_{j+1}^3+3A_j^2 A_{j+1})\cos (\varDelta _{j+1})\nonumber \\&\quad -A_j A_{j+1}^2\cos (2\varDelta _{j+1})+(A_{j-1}^3+3A_j^2 A_{j-1})\cos (\varDelta _{j})\nonumber \\&\quad -A_j A_{j-1}^2\cos (2\varDelta _{j})-2(A_j^3+A_j A_{j+1}^2+A_j A_{j-1}^2)\bigg ]\nonumber \\&\quad +\frac{\varGamma }{4\omega _0^2 A_j}+\zeta _j \end{aligned}$$

and

68 $$\begin{aligned}&{\dot{\varphi }}_j=\frac{\lambda }{2}\left[ \frac{A_{j+1}}{A_j}\sin (\varDelta _{j+1})-\frac{A_{j-1}}{A_j}\sin (\varDelta _{j})\right] \nonumber \\&\quad +\frac{\vartheta }{8}\bigg [\Big (A_j A_{j+1}+\frac{A_{j+1}^3}{A_j}\Big )\sin (\varDelta _{j+1})-A_{j+1}^2\nonumber \\&\quad \sin (2\varDelta _{j+1})-\Big (A_j A_{j-1}+\frac{A_{j-1}^3}{A_j}\Big )\sin (\varDelta _{j})\nonumber \\&\quad +A_{j-1}^2\sin (2\varDelta _{j})\bigg ]+\frac{\chi _j}{A_j}, \end{aligned}$$

for $$j=1,\ldots ,N$$.

### Derivation of the solution of the stationary Fokker–Planck equation

Below, we derive the solution ${\mathcal {P}}^\infty ({\varvec{z}})={\mathcal {P}}({\varvec{z}},t\rightarrow \infty )$, where ${\varvec{z}}=(\eta _1,{\dot{\eta }}_1,\ldots ,\eta _N,{\dot{\eta }}_N)$, of the stationary Fokker–Planck equation (31) associated with the slow-flow system (5) and (6). Our derivation is based on an understanding of the transformation properties of the stationary Fokker–Planck equation under an invertible mapping ${\varvec{f}}$ from rectilinear coordinates ${\varvec{x}}$ to curvilinear coordinates ${\varvec{y}}$:

69 $$\begin{aligned} {\varvec{x}}={\varvec{f}}({\varvec{y}}). \end{aligned}$$

We assume that the Jacobian $\varvec{J_f}=\partial {\varvec{f}}/\partial {\varvec{y}}$ is invertible and can be written as

70 $$\begin{aligned} \varvec{J_f}({\varvec{y}})={\varvec{Q}}({\varvec{y}}){\varvec{h}}({\varvec{y}}), \end{aligned}$$

where ${\varvec{Q}}={\varvec{Q}}^{-T}$ is an orthogonal rotation matrix with $|\mathrm {det}({\varvec{Q}})|=1$ and ${\varvec{h}}$ is a diagonal matrix. This implies ${\varvec{h}}$ is related to the metric tensor ${\varvec{g}}=\varvec{J_f}^T\varvec{J_f}$ by

71 $$\begin{aligned} {\varvec{h}}^T({\varvec{y}}) {\varvec{h}}({\varvec{y}}) ={\varvec{g}}({\varvec{y}}). \end{aligned}$$

Now consider a stochastically perturbed gradient system, given by the following Langevin equation in rectilinear coordinates ${\varvec{x}}$:

72 $$\begin{aligned} \dot{{\varvec{x}}}=-\frac{\partial {\mathcal {V}}}{\partial {\varvec{x}}}+{\varvec{n}}, \end{aligned}$$

where ${\varvec{n}}$ contains additive white noise components in the directions of the state variables ${\varvec{x}}$, which are all assumed to have the same noise intensity $\varGamma /2\omega _0^2$. For a system of the type of Eq. (72), the stationary Fokker–Planck equation [95] is given by Eq. (31), which, for ${\mathcal {P}}^\infty \ne 0$, has the following normalizable solution:

73 $$\begin{aligned} {\mathcal {P}}^\infty ({\varvec{x}})={\mathcal {N}} \exp {(-4\omega _0^2 {\mathcal {V}}({\varvec{x}})/\varGamma )}, \end{aligned}$$

where ${\mathcal {N}}\in {\mathbb {R}}$ is a normalization constant. In ${\varvec{y}}$-coordinates, the probability of the stationary state of the system being within some domain ${\varvec{f}}^{-1}({\mathcal {D}})$ is given by

74 $$\begin{aligned} P=\int _{{\varvec{f}}^{-1}({\mathcal {D}})} {{\mathcal {P}}^\infty }({\varvec{f}}({\varvec{y}})) |\mathrm {det}({\varvec{h}})| d{\widetilde{V}}, \end{aligned}$$

where the fact that $|\mathrm {det}(\varvec{J_f})|=|\mathrm {det}({\varvec{h}})|$ was used. With this, the stationary PDF reads, in ${\varvec{y}}$-coordinates,

75 $$\begin{aligned} {\mathcal {P}}^\infty ({\varvec{y}})= & {} |\mathrm {det}({\varvec{h}})|{\mathcal {N}} \exp {(-4\omega _0^2 {\mathcal {V}}({\varvec{f}}({\varvec{y}}))/\varGamma )} \nonumber \\= & {} |\mathrm {det}({\varvec{h}})|{\mathcal {N}} \exp {(-4\omega _0^2 {\mathcal {V}}({\varvec{x}})/\varGamma )}\nonumber \\= & {} |\mathrm {det}({\varvec{h}})|{\mathcal {P}}^\infty ({\varvec{x}}). \end{aligned}$$

Under the mapping (69), the Langevin equation (72) becomes

76 $$\begin{aligned} \dot{{\varvec{y}}}=-{\varvec{g}}^{-1}({\varvec{y}})\frac{\mathrm {d}{\mathcal {V}}({\varvec{y}})}{\mathrm {d} {\varvec{y}}}+{\varvec{h}}^{-1}({\varvec{y}})\tilde{{\varvec{n}}}, \end{aligned}$$

where $\tilde{{\varvec{n}}}={\varvec{Q}}^T {\varvec{n}}$ contains the noise components in the direction of the ${\varvec{y}}$-coordinates. The solution of the stationary Fokker–Planck equation ${\mathcal {P}}^\infty ({\varvec{y}})$ associated with the transformed Langevin equation (76) is given by Eq. (75).

In the present case, the rectilinear coordinates ${\varvec{x}}$, the curvilinear coordinates ${\varvec{y}}$ and the mapping ${\varvec{f}}({\varvec{y}})$ are given by

77 $$\begin{aligned} {\varvec{x}}= & {} (U_1,V_1,\ldots ,U_N,V_N), \end{aligned}$$

78 $$\begin{aligned} {\varvec{y}}= & {} (A_1,\varphi _1,\ldots ,A_N,\varphi _N), \end{aligned}$$

79 $$\begin{aligned} {\varvec{f}}({\varvec{y}})= & {} [A_1 \cos (\varphi _1), A_1 \sin (\varphi _1),\ldots ,\nonumber \\&\quad A_N \cos (\varphi _N), A_N\sin (\varphi _N)]. \end{aligned}$$

The Jacobian of the mapping ${\varvec{f}}$ reads

80 $$\begin{aligned} \varvec{J_f}= \begin{pmatrix} {\varvec{M}}_1 &{} {\varvec{0}} &{}\ldots &{}{\varvec{0}}&{}{\varvec{0}} \\ {\varvec{0}} &{} {\varvec{M}}_2 &{} \ldots &{}{\varvec{0}}&{} {\varvec{0}} \\ \ldots &{} \ldots &{}\ldots &{}\ldots &{}\ldots \\ {\varvec{0}} &{} {\varvec{0}} &{} \ldots &{} {\varvec{M}}_{N-1} &{} {\varvec{0}} \\ {\varvec{0}} &{} {\varvec{0}} &{} \ldots &{}{\varvec{0}} &{} {\varvec{M}}_{N} \end{pmatrix}{\varvec{h}}, \end{aligned}$$

where $${\varvec{h}}=\mathrm {diag}(1,A_1,\ldots ,1,A_N)$$ and

81 $$\begin{aligned} {\varvec{M}}_j= \begin{pmatrix} \cos (\varphi _j) &{} -\sin (\varphi _j) \\ \sin (\varphi _j) &{} \cos (\varphi _j)\\ \end{pmatrix}. \end{aligned}$$

The matrix ${\varvec{Q}}=\varvec{J_f}\,{\varvec{h}}^{-1}$ is a block-diagonal matrix of orthogonal matrices, and is thus itself orthogonal. Hence, Eq. (70) is satisfied for the present system.

To derive the solution of the stationary Fokker–Planck equation (31), we note that the slow-flow system (5) and (6) is equivalent to a Langevin equation of the form given in Eq. (76), where ${\varvec{y}}=(A_1,\varphi _1,\ldots ,A_N,\varphi _N)$,

82 $$\begin{aligned} {\varvec{h}}^{-1}= & {} \mathrm {diag}(1,A_1^{-1},\ldots ,1,A_N^{-1}) \in {\mathbb {R}}^{{2N}\times {2N}}, \end{aligned}$$

83 $$\begin{aligned} {\varvec{g}}^{-1}= & {} {\varvec{h}}^{-1} {\varvec{h}}^{-T}\nonumber \\= & {} \mathrm {diag}(1, A_1^{-2},\ldots ,1,A_N^{-2})\in {\mathbb {R}}^{{2N}\times {2N}}, \end{aligned}$$

and the potential ${\mathcal {V}}$ is defined by Eq. (9). We find from Eq. (82) that

84 $$\begin{aligned} \mathrm {det}({\varvec{h}})=\prod ^N_{j=1} A_j. \end{aligned}$$

With this result, the stationary solution of the Fokker–Planck equation associated with the slow-flow system (5) and (6) can be directly computed from Eq. (75):

85 $$\begin{aligned}&{\mathcal {P}}^\infty (A_m, \varphi _m)\nonumber \\&\quad ={\mathcal {N}}\Big [\prod ^N_{j=1} A_j\Big ] \exp {(-4\omega _0^2 {\mathcal {V}}(A_m, \varphi _m)/\varGamma )}, \end{aligned}$$

$m=1,\ldots ,N$. Combining Eqs. (75) and (85) yields the stationary PDF in ${\varvec{x}}$-coordinates (see Eq. (77)):

86 $$\begin{aligned}&{\mathcal {P}}^\infty (U_m, V_m) \nonumber \\&\quad ={\mathcal {N}} \exp {(-4\omega _0^2 {\mathcal {V}}(A_m(U_m,V_m), \varphi _m(U_m,V_m))/\varGamma )}, \nonumber \\ \end{aligned}$$

$$m=1,\ldots ,N$$, where

87 $$\begin{aligned} A_m(U_m,V_m)= & {} \sqrt{U_m^2+V_m^2}, \end{aligned}$$

88 $$\begin{aligned} \varphi _m(U_m,V_m)= & {} \arctan (V_m,U_m). \end{aligned}$$

We now consider the mapping ${\varvec{z}}={\varvec{d}}({\varvec{x}})$ from ${\varvec{x}}$ to the fast ${\varvec{z}}$ coordinates given by

89 $$\begin{aligned} {\varvec{z}}= & {} (\eta _1,{\dot{\eta }}_1,\ldots ,\eta _N,{\dot{\eta }}_N), \end{aligned}$$

90 $$\begin{aligned} {\varvec{x}}= & {} (U_1,V_1,\ldots ,U_N,V_N), \end{aligned}$$

91 $$\begin{aligned} {\varvec{d}}({\varvec{x}})= & {} [A_1 \cos (\phi _1),-\omega _0 A_1 \sin (\phi _1),\ldots ,\nonumber \\&\quad A_N \cos (\phi _N),-\omega _0 A_N\sin (\phi _N)], \end{aligned}$$

where $\phi _j=\varphi _j+\omega _0 t$ and the amplitude-phase coordinates ($A_j$, $\varphi _j$) are expressed in terms of $U_j$ and $V_j$ using Eqs. (87) and (88), respectively. The Jacobian of the mapping ${\varvec{d}}$ reads

92 $$\begin{aligned}&\varvec{J_d}= \begin{pmatrix} {\varvec{H}}_1 &{} {\varvec{0}} &{}\ldots &{}{\varvec{0}}&{}{\varvec{0}} \\ {\varvec{0}} &{} {\varvec{H}}_2 &{} \ldots &{}{\varvec{0}}&{} {\varvec{0}} \\ \ldots &{} \ldots &{}\ldots &{}\ldots &{}\ldots \\ {\varvec{0}} &{} {\varvec{0}} &{} \ldots &{} {\varvec{H}}_{N-1} &{} {\varvec{0}} \\ {\varvec{0}} &{} {\varvec{0}} &{} \ldots &{}{\varvec{0}} &{} {\varvec{H}}_{N} \end{pmatrix}, \end{aligned}$$

where

93 $$\begin{aligned}&{\varvec{H}}_j= \frac{1}{\sqrt{U_j^2+V_j^2}}\begin{pmatrix} H_{11}(U_j,V_j)&{} H_{12}(V_j,U_j) \\ H_{21}(U_j,V_j) &{} H_{22}(V_j,U_j)\\ \end{pmatrix},\nonumber \\ \end{aligned}$$

94 $$\begin{aligned}&H_{11}(U_j,V_j)=U_j\cos (\phi _j) + V_j\sin (\phi _j), \end{aligned}$$

95 $$\begin{aligned}&H_{12}(U_j,V_j)=V_j\cos (\phi _j) - U_j\sin (\phi _j), \end{aligned}$$

96 $$\begin{aligned}&H_{21}(U_j,V_j)=-\omega _0\big [U_j\sin (\phi _j) - V_j\cos (\phi _j)\big ],\nonumber \\ \end{aligned}$$

97 $$\begin{aligned}&H_{22}(U_j,V_j)=-\omega _0\big [V_j\sin (\phi _j) + U_j\cos (\phi _j)\big ],\nonumber \\ \end{aligned}$$

and the dependence of $\phi _j$ on $U_j$ and $V_j$ is suppressed for compactness. The modulus of the determinant of $\varvec{J_d}$ is the positive real constant $|\det (\varvec{J_d})|=\omega _0^{N}$, which is absorbed into the normalization factor ${\mathcal {N}}$. Since

98 $$\begin{aligned} {\mathcal {P}}^\infty ({\varvec{z}})=|\mathrm {det}(\varvec{J_d}) |{\mathcal {P}}^\infty ({\varvec{x}}) \end{aligned}$$

and by the definition of ${\mathcal {P}}^\infty ({\varvec{x}})$ in Eq. (86), the stationary PDF of the fast system (2) is given by

99 $$\begin{aligned}&{\mathcal {P}}^\infty (\eta _m, {\dot{\eta }}_m) \nonumber \\&\quad ={\mathcal {N}} \exp {(-4\omega _0^2 {\mathcal {V}}(A_m(\eta _m,{\dot{\eta }}_m), \varphi _m(\eta _m,{\dot{\eta }}_m))/\varGamma )}, \nonumber \\ \end{aligned}$$

$$m=1,\ldots ,N$$, where

100 $$\begin{aligned} A_m(\eta _m,{\dot{\eta }}_m)= & {} \sqrt{\eta _m^2+\left( {\dot{\eta }}_m/\omega _0\right) ^2}, \end{aligned}$$

101 $$\begin{aligned} \varphi _m(\eta _m,{\dot{\eta }}_m)= & {} -\arctan ({\dot{\eta }}_m/\omega _0,\eta _m)-\omega _0 t, \end{aligned}$$

the potential ${\mathcal {V}}$ is given by Eq. (9) and ${\mathcal {N}}$ is a normalization constant.

## Declarations

### Supplementary material

Animated examples of time traces of $A_j$, $\varDelta _j$, $\varphi _j$ and $\eta _j$, computed from the noise-free ($G=0$) and noise-driven ($G=2.88\times 10^{-1}$) slow-flow system (5) and (6) over five acoustic cycles in the steady state for different values of $\varLambda$, are included in the supplementary material. For visualization purposes, the color bar of the time traces of $\eta _j$ in the noise-driven case is cut off at $\pm 1.2 A_0^*$. As in Fig. 17, in the plots showing the distribution of the acoustic pressure $\eta _j$ along the ring, the radii of the small circles scale with $|\eta _j|$.
